# A Hierarchical Multimodal Knowledge Graph for Neural Cell-Type-Specific Regulation Integrating Single-Cell Transcriptomics and Literature Evidence

**DOI:** 10.3390/ijms27156842

**Published:** 2026-07-30

**Authors:** Chuangyu Chen, Xiaomin Ni, Yang Min, Zhen Wang, Zhilan Xu, Yang Zhang, Hao Yu

**Affiliations:** 1 Institute of Biomedical and Health Engineering, Shenzhen Institute of Advanced Technology, Chinese Academy of Sciences, Shenzhen 518055, China; chenchuangyu25@mails.ucas.ac.cn (C.C.); 18110700109@fudan.edu.cn (X.N.); y.m@siat.ac.cn (Y.M.); zhen.wang@hotmail.fr (Z.W.); xuzhilan_q2@163.com (Z.X.); 2 School of Life Sciences, University of Chinese Academy of Sciences, Beijing 100049, China; 3 Institute of Molecular Physiology, Shenzhen Bay Laboratory, Shenzhen 518132, China; 4 The Key Laboratory of Biomedical Imaging Science and System, Chinese Academy of Sciences, Shenzhen 518055, China

**Keywords:** neural cell types, knowledge graph, single-cell transcriptomics, literature mining, knowledge graph embedding, link prediction

## Abstract

The nervous system comprises highly diverse cell types governed by cell-type-specific molecular regulatory programs. However, regulatory evidence is scattered across unstructured literature and described using inconsistent cell-type nomenclature and granularity, hindering systematic integration and cross-study comparison. Here, we construct a neural-cell-centric multimodal knowledge graph that transforms fragmented regulatory evidence into a standardized, computable substrate. We establish a three-level hierarchical cell-type taxonomy anchored to the Cell Ontology (79 nodes), integrate two large-scale human brain single-cell transcriptomic datasets (over 4 million cells) to derive molecular fingerprints, and use a large language model to retain 25,812 curated regulatory evidence records from PubMed abstracts. The resulting Neo4j graph contains 41,532 directed relationships. For knowledge graph embedding, we export a deduplicated non-paper training subgraph containing 19,819 triples over 10,660 entities, supporting cell-type-specific link prediction that prioritizes candidate regulators and markers, illustrated here for microglia. This framework provides a structured basis for cross-study comparison, hypothesis generation and knowledge-guided reasoning in neural cell-type-specific regulation.

## 1. Introduction

The nervous system comprises highly diverse cell types governed by complex regulatory programs [1,2]. The same regulatory factor may exert distinct or even opposing effects across neural cell types, making cell-type specificity essential for understanding neural circuit organization, functional maintenance and disease mechanisms. Context-dependent regulatory effects are widely observed across diverse biomedical systems. However, evidence for cell-type-specific regulation is dispersed across studies that often use inconsistent cell-type nomenclature, granularity and hierarchical definitions. Therefore, a stable and comparable cell-type reference framework is required to systematically organize neural regulatory knowledge. Knowledge graphs provide a suitable representation for this task by encoding heterogeneous biomedical entities and their relationships within a unified semantic structure.

Single-cell transcriptomics has provided a data foundation for building such a framework by enabling molecular definitions of cell identity [1,2,3,4]. However, a large body of regulatory evidence remains scattered across the unstructured biomedical literature, where cell-type terms often fail to align with high-resolution transcriptomic classifications. This mismatch makes it difficult to reliably map literature evidence to molecularly defined cell types and limits cross-study comparison. Knowledge graphs can bridge this gap by mapping heterogeneous, multi-granularity data into a unified semantic space through standardized entity identifiers and relation types [5].

From a knowledge representation perspective, single-cell transcriptomics answers “which cell is it?” by providing molecular fingerprints, whereas the literature evidence answers “which factor acts on which cell and with what effect?”, that is, perturbation-response knowledge. The semantic and evidential gap between these two information types represents a major challenge for integrating neural regulatory knowledge. Existing biomedical knowledge graphs are mostly centered on genes, diseases or drugs; they lack a hierarchical, cell-type-centric structure and have not systematically incorporated single-cell molecular fingerprints together with literature-derived regulatory evidence [6,7]. Constructing a hierarchical, multimodal knowledge graph centered on neural cell types is therefore a practical route to unify molecular fingerprints and regulatory evidence.

To address these challenges, we constructed a three-level hierarchical neural cell knowledge graph. Using an expert-guided pruning strategy, we unified cell-type representation and integrated single-cell transcriptomic fingerprints with literature-derived regulatory evidence from PubMed. The resulting framework supports cross-source, cross-granularity knowledge integration and provides a computable basis for predicting cell-type-specific regulatory relationships, retrieving marker genes and discovering putative regulatory pathways.

## 2. Results

### 2.1. A Hierarchical Neural Cell Type Taxonomy for Knowledge Graph Construction

**Figure 1:**
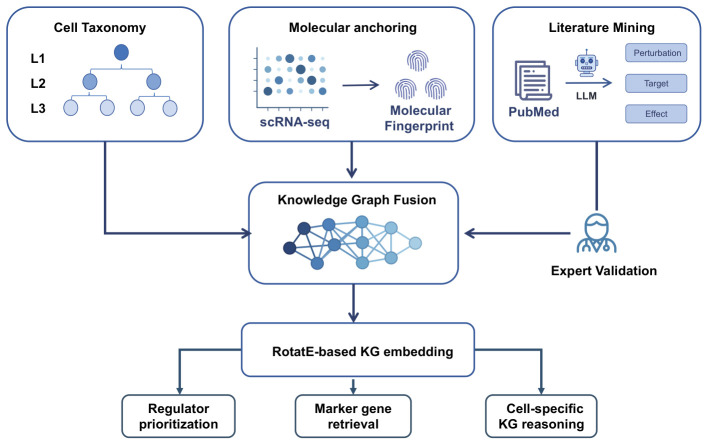
Framework for constructing a multimodal neural cell knowledge graph. The workflow combines a Cell Ontology-guided three-level neural cell hierarchy, scRNA-seq-derived molecular fingerprints, and LLM-extracted PubMed regulatory triples into a Neo4j-based knowledge graph, followed by knowledge graph embedding for downstream prediction tasks.

We built a three-level neural cell type taxonomy using the Cell Ontology as the primary semantic backbone [8,9,10] and refined it with cell-type annotations from two human brain single-cell transcriptomic atlases [11,12]. The hierarchy contains 79 core nodes (Figure 1; Supplemental Table S1). Level 1 divides cells into four major branches—Neuron, Glial cell, Progenitor and Non-neural cell of the nervous system—covering the neural lineage from a developmental perspective. Level 2 converges redundant Cell Ontology branches into 30 functional units with clear physiological significance, including excitatory neurons, inhibitory neurons, astrocytes and meningeal cells. Level 3 resolves transcriptomic heterogeneity by incorporating 45 high-resolution subtypes, such as hippocampal CA1, CA2 and CA3 pyramidal neurons, chandelier cells and anatomically localized vascular endothelial progenitor cells.

This hierarchy provides standardized semantic anchors for mapping literature-derived evidence across studies. Importantly, it establishes a traceable path from coarse to fine granularity. Even when a study reports only a broad term such as pyramidal neuron, the hierarchy allows its association with more precise subtypes while preserving the original evidence level. Each cell type node incorporates a textual definition and all definitions are traced to their original sources.

To ensure the hierarchy is traceable and reusable, each node was assigned a unique identifier, canonical name, level, parent node, definition, evidence source, available Cell Ontology identifier, and molecular-anchor status. Identical or similar names across levels were disambiguated by node ID, level, and parent assignment, preserving fine-grained literature semantics while distinguishing direct molecular anchors from propagated context and semantic-only nodes. The complete 79-node hierarchy and annotations are provided in Supplemental Table S1.

### 2.2. Molecular Fingerprints for Cell Types from Single-Cell Transcriptomics

To link the literature-based cell type taxonomy to molecular identities defined by transcriptomics, we integrated two published human brain single-cell RNA-seq datasets as a steady-state reference: an adult human brain atlas (2.37 million cells) and an early developmental atlas (1.78 million cells), totaling 4.16 million cells and approximately 59,000 genes [11,12].

**Figure 2:**
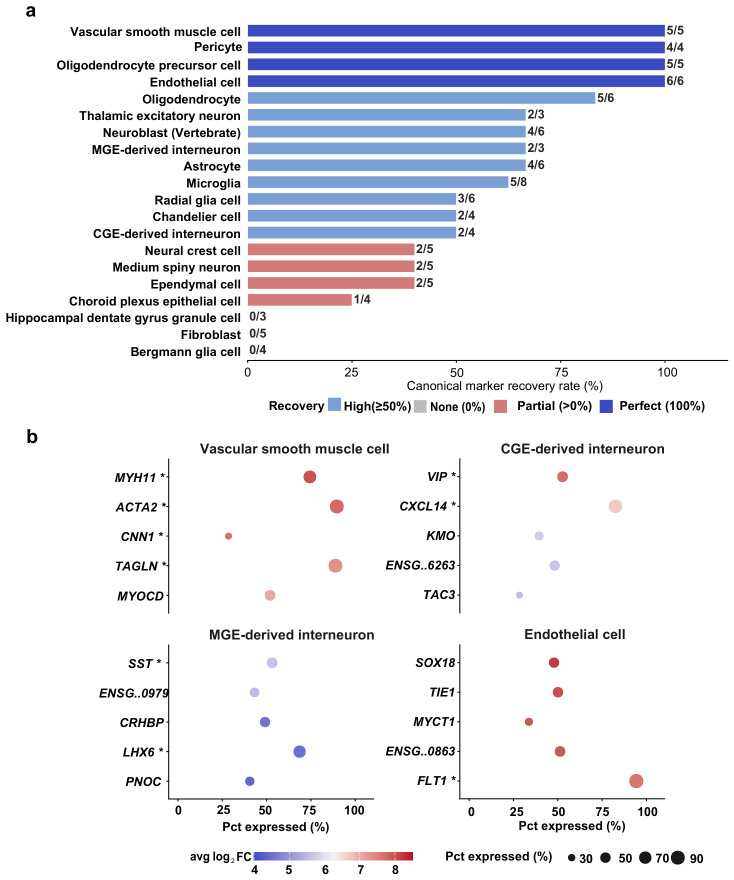
Cell-type-specific marker gene validation from single-cell transcriptomic data: (**a**) Recovery of classic marker genes across representative cell types. Bars show the proportion of known classic markers for each cell type that appeared in the corresponding Top 50 candidate marker list. Colors indicate complete (100%), high (≥50%), partial (>0%) or no (0%) recovery. The 50% threshold is a visualization categorization aid that partitions cell types into roughly balanced color groups for readability; it is not a statistical cutoff and does not affect quantitative interpretation of recovery rates. (**b**) Expression patterns of the top five marker genes in four selected representative cell types. Bubble position and size indicate the percentage of cells expressing each gene within the target cell type; color indicates differential expression strength, shown on a scale matched to the displayed representative markers. Asterisks indicate top-ranked markers that were also recovered classic markers.

For each cell type in our taxonomy, we derived a set of candidate marker genes showing strong differential expression and cell-type specificity. Cross-validation with the CellMarker database [13] showed that across 20 cell types and 97 classic markers 56 appeared in the corresponding Top 50 candidate lists, giving an overall hit rate of 57.7% (Figure 2a,b; Supplemental Tables S2 and S3). Representative examples include MYH11 in vascular smooth muscle cells, FLT1 in endothelial cells and PDGFRA in oligodendrocyte precursor cells, all of which ranked highly in their respective Top 50 lists. These genes are well established in smooth muscle contractile phenotype maintenance, angiogenesis regulation and OPC lineage specification, respectively [14,15,16], supporting the biological validity of the molecular fingerprinting framework.

To contextualize this overall recovery rate, we further stratified marker recovery by cell type. Recovery was heterogeneous across the 20 evaluated cell types. Four cell types, including vascular smooth muscle cell, pericyte, oligodendrocyte precursor cell and endothelial cell, showed complete recovery of CellMarker-listed classic markers. Nine additional cell types recovered at least half of the evaluated markers, whereas four cell types showed partial recovery and three cell types showed no recovery under the strict Top 50 criterion, including Bergmann glia cell, fibroblast and hippocampal dentate gyrus granule cell. Therefore, the 57.7% overall recovery rate should be interpreted as partial external concordance with CellMarker 2.0 rather than complete recovery of all known canonical markers.

**Figure 3:**
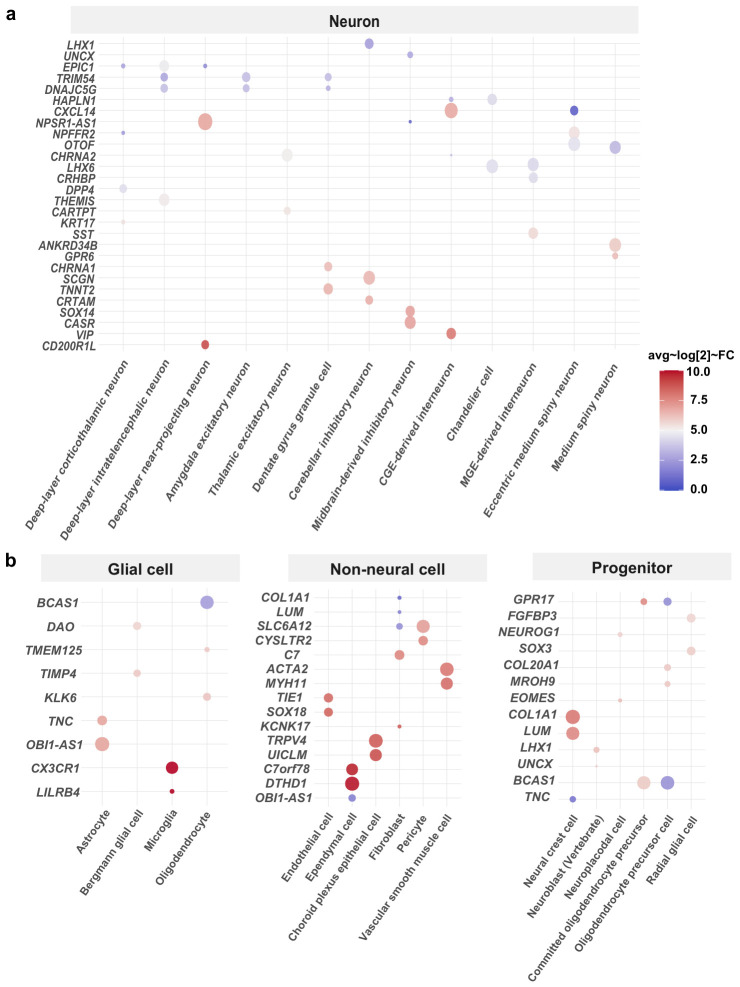
Molecular fingerprint atlas of neural cell subtypes based on marker gene expression patterns. The dot plot uses dot color to indicate average log_2_ fold change and dot size to indicate the proportion of cells expressing each gene. (**a**) Marker gene expression across neuron subtypes, grouped by neuronal lineages. (**b**) Marker gene expression across non-neuron lineages, including glial cells, non-neural cells, and progenitors, grouped by Level 1 lineage.

The granularity of the cell types in the knowledge graph does not always match that of the reference transcriptome annotations. Where transcriptome data did not further resolve a cell population, we retained fine-grained subtypes in the knowledge graph to accommodate the literature evidence, but we built molecular anchors only at the upper nodes stably supported by the transcriptome data. These molecular features were then propagated to child nodes via the hierarchical relationship. This design enabled robust hierarchical alignment between text-based and transcriptome-based evidence. The final molecular fingerprint atlas covers 31 neural cell subtypes (Figure 3).

### 2.3. Neural Regulatory Evidence Extraction from the Literature Using a Large Language Model

We used a large language model to systematically extract neural regulatory evidence from PubMed abstracts [17]. From 1,160,742 candidate abstracts, the pipeline yielded 25,812 retained evidence records after LLM-based screening, information extraction and manual curation. These records cover regulatory effects of transcription factors, receptors, signaling pathways, drug treatments and other perturbations on gene expression, cell activation state, morphology and survival across L1, L2 and L3 cell types. Most retained L2-level records received high internal confidence scores from the extraction pipeline: 97.5% had a score ≥8 on a 0–10 scale. Confidence scores were similarly high across the other two levels, with 97.4% of L1 records and 94.8% of L3 records also scoring ≥8, indicating that extraction confidence remained consistently high even for the more heterogeneous fine-grained subtypes that constitute the majority of REGULATES relationships. These scores were used for prioritization and quality control, whereas extraction accuracy was evaluated separately by expert review.

**Figure 4:**
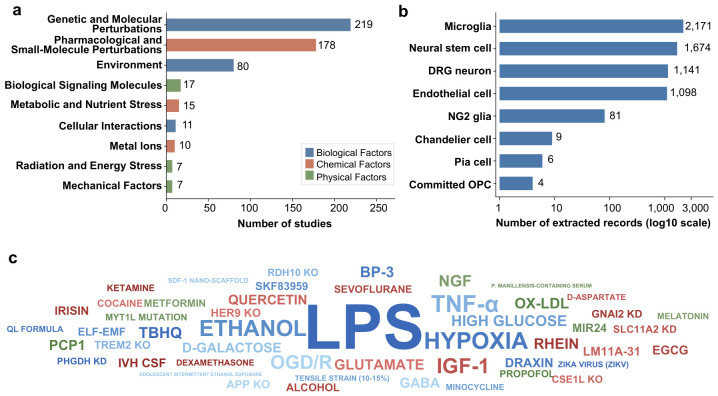
Literature-derived neural regulatory triples across cell types and perturbation categories: (**a**) Long-tail distribution of extracted triples across representative L3 cell types. The horizontal axis is log10-scaled. Microglia and neural stem cells show the highest counts, whereas pia cells and committed OPCs show the lowest. (**b**) Hierarchical distribution of perturbation factors at L1 level. Genetic/molecular perturbations and pharmacological/small-molecule perturbations dominate. (**c**) Word cloud of perturbagens for L3 cell types. Font size is proportional to frequency. High-frequency terms include LPS, ethanol, TNF-α, hypoxia, and OGD/R.

The extracted evidence exhibited a pronounced long-tail distribution across cell types (Figure 4a). Microglia and neural stem cells had the highest number of regulatory records, reflecting concentrated research efforts on neuroinflammation and adult neurogenesis. Dorsal root ganglion neurons were also highly represented, consistent with their established role in pain signaling, and endothelial cells showed substantial coverage due to ongoing interest in blood–brain barrier regulation. By contrast, highly specialized cell types such as committed oligodendrocyte precursor cells, pia cells and chandelier cells had few records, indicating that these cell types remain under-characterized in the existing literature.

Analysis of perturbation types (Figure 4b) showed that genetic and molecular perturbations, together with pharmacological and small-molecule perturbations, accounted for the majority of the records. Biological signaling molecules formed the third largest category, highlighting the importance of cell–cell communication mechanisms. The word cloud of high-frequency perturbagens (Figure 4c) revealed that LPS, ethanol, TNF-α, hypoxia and OGD/R dominated the records, pointing to inflammation, metabolic stress and ischemic injury as predominant experimental contexts in cell-type-specific neuroregulation research.

To validate extraction accuracy, we sampled 331 triples spanning six cell types: 100 dopaminergic neurons (L2), 100 microglia (L3), 50 oligodendrocyte precursor cells (L3), 50 dorsal raphe serotonergic neurons (L3), 23 chandelier cells (L3) and 8 pia cells (L3). Two domain experts independently evaluated these 331 triples against a predefined gold standard. The overall extraction accuracy was 96.4% (319/331), with 92.0% for dopaminergic neurons, 98.0% for microglia, 100.0% for oligodendrocyte precursor cells, 100.0% for dorsal raphe serotonergic neurons, 91.3% for chandelier cells and 100.0% for pia cells. Dimension-specific accuracies were 99.4% for entity recognition, 98.8% for cell type mapping and 98.5% for relation logic. Inter-annotator agreement was 99.7% (330/331) with Cohen’s κ=0.955. Error analysis identified relation logic errors as the most frequent (45.5% of errors), followed by cell type mapping errors (36.4%) and entity recognition errors (18.2%). Detailed inter-annotator agreement and error distribution are provided in Supplemental Table S4.

Qualitative analysis of the five relation-logic errors (Type C) revealed three recurrent patterns: (i) direction reversals, where effect_direction contradicted textual semantics (2/5; e.g., MPTP-induced neurodegeneration encoded as upregulation, PMID 41360225); (ii) causal-polarity confusion in protective contexts (1/5; LAG3 knockout preserving tyrosine hydroxylase, PMID 41542562); and (iii) non-regulatory events misclassified as REGULATES edges (2/5; an acute electrophysiological response, PMID 8035215, and a downstream phosphorylation cascade, PMID 37922650). The errors spanned chemical, genetic, microbiome and physical perturbations without concentration on a single factor class, but they showed weak enrichment in dopaminergic-neuron contexts (3/5). All five validated error cases were corrected during manual curation by reversing direction, reclassifying the relationship type, or removing the record from the REGULATES set. These corrections reduced the risk that the identified failure patterns propagated into the final REGULATES set, although residual relation-logic errors may remain in the full graph (Supplemental Table S4b).

### 2.4. Mapping Molecular Fingerprints and Literature Evidence onto the Hierarchical Backbone

**Figure 5:**
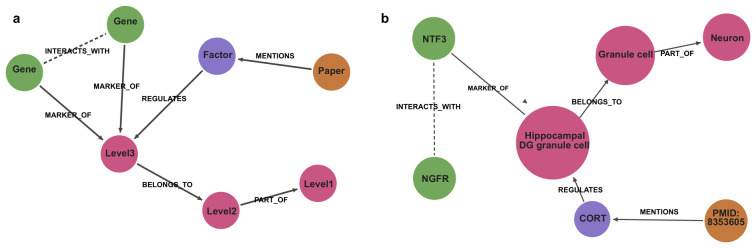
Structure and example of the neural cell knowledge graph: (**a**) Graph schema showing the main node and edge types. Cell nodes are organized by BELONGS_TO and PART_OF relationships; genes, factors and papers are linked through MARKER_OF, REGULATES, INTERACTS_WITH and MENTIONS edges. (**b**) Example subgraph centered on the Level-3 cell type Hippocampal dentate gyrus granule cell. The subgraph shows how transcriptional marker NTF3, perturbation factor CORT, STRING-derived NTF3–NGFR interaction and PubMed evidence are integrated around the same fine-grained cell-type anchor within the L3–L2–L1 hierarchy.

Integration of the three-level neural cell type hierarchy, molecular fingerprints and literature-derived regulatory evidence into Neo4j yielded a unified multimodal neural cell knowledge graph (Figure 5). The complete Neo4j graph contains 18,609 nodes and 41,532 directed relationships across six major node types: Level 1, Level 2 and Level 3 cell types, genes, regulatory factors and PubMed papers. These nodes include 9782 regulatory factors, 7463 PubMed papers, 1285 genes and 79 cell type nodes. The hierarchical organization of cell types is captured by 45 BELONGS_TO edges linking Level 3 nodes to Level 2 nodes and 30 PART_OF edges linking Level 2 nodes to Level 1 nodes. Detailed topological analysis of the knowledge graph, including node degree distribution, relationship type distribution and hub node identification, is provided in Supplemental Figure S1.

The literature-mining pipeline produced 25,812 retained regulatory evidence records across Level 1, Level 2 and Level 3 cell types after LLM-based extraction and quality control. In the complete Neo4j graph, these records are represented as 25,202 REGULATES relationships after graph import and relationship merging, distributed across Level 1 (527), Level 2 (6404) and Level 3 (18,271) targets. PubMed source papers are retained as Paper nodes and linked to regulatory factors by 12,349 MENTIONS relationships for evidence traceability. For KGE training, Paper nodes and MENTIONS relationships were excluded, and a deduplicated non-paper subgraph was exported. The reduction from 18,609 nodes in the complete Neo4j graph to 10,660 entities in the KGE subgraph primarily reflects the exclusion of 7463 Paper nodes (retained only for evidence traceability); the remaining difference corresponds to entities that do not participate in any retained relationship triple after MENTIONS removal. The final KGE input subgraph contains 19,819 triples over 10,660 entities: 12,494 REGULATES, 2310 MARKER_OF, 4940 STRING-derived INTERACTS_WITH, 45 BELONGS_TO and 30 PART_OF triples (Supplemental Table S5).

### 2.5. Cell-Type-Specific Regulatory Relationship Prediction via Knowledge Graph Embedding

To test whether our knowledge graph can generate biologically meaningful hypotheses, we used link prediction for REGULATES edges to prioritize unseen cell–perturbagen relationships. Entities (Cell, Factor and Gene) and relations (REGULATES, MARKER_OF and INTERACTS_WITH) were jointly embedded into a low-dimensional space, allowing the model to integrate cell hierarchy, molecular markers, literature evidence and gene functional interactions.

**Figure 6:**
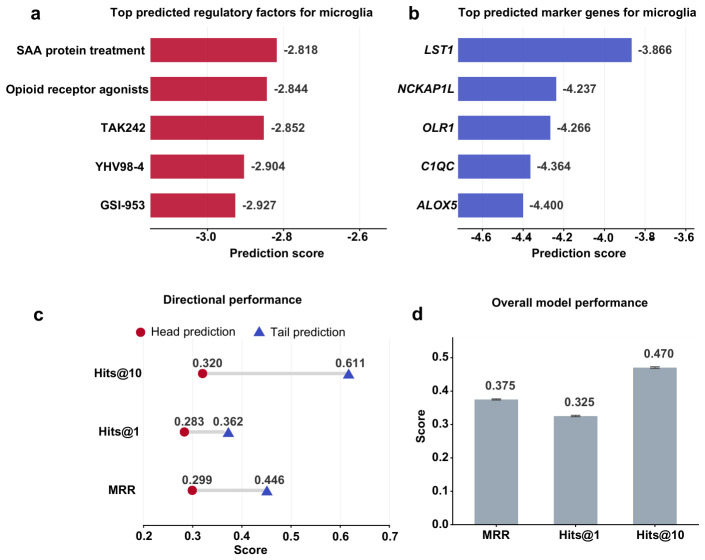
Knowledge graph embedding-based prediction of cell-type-specific regulatory relationships: (**a**) Top-5 upstream regulator predictions for L3:Microglia. The horizontal axis represents the prediction score, where values closer to zero indicate higher rank, and the vertical axis lists candidate regulator names. (**b**) Top-5 marker gene predictions for L3:Microglia. The horizontal axis represents the prediction score, and the vertical axis lists candidate gene names. (**c**) Directional asymmetry analysis of REGULATES prediction in the seed-42 RotatE model. Head prediction (red circles) fixes the target cell type and ranks candidate upstream Factor entities; tail prediction (blue triangles) fixes the Factor entity and ranks candidate target cell types. (**d**) Overall filtered link-prediction performance of the RotatE model across three random seeds. Bars show mean values and error bars show s.e.m. The green annotation highlights L3:Microglia-specific tail-ranking performance in the seed-42 REGULATES evaluation.

Taking microglia (L3:Microglia) as an example, the trained RotatE model (Supplemental Table S6; Supplemental Figure S2) ranked SAA protein treatment, opioid receptor agonists, TAK242, YHV98-4 and GSI-953 among the top candidates (Figure 6a). These are known to be involved in neuroinflammation, TLR4 signaling and neurodegeneration [18,19,20,21], suggesting that the embedding captures microglia-relevant biology. Concurrently, the model identified LST1, NCKAP1L and OLR1 as top marker gene prediction (Figure 6b), confirming that it preserves microglial molecular identity.

Bidirectional prediction was strongly asymmetric (Figure 6c; Supplemental Table S7). Head prediction (cell type → upstream regulator) gave MRR = 0.299 and Hits@10 = 0.320, whereas tail prediction (regulator → cell type) achieved MRR = 0.446 and Hits@10 = 0.611. Because head prediction is inherently more difficult but matches our use case, we adopted a type-restricted scoring strategy for practical hypothesis generation.

To assess the biological plausibility of candidate regulators that were not present in the training graph but were prioritized by KGE, we selected three examples—FMR1, PTEN and FKBP5—none of which had a direct REGULATES edge to microglia in the training graph, though each has independent literature support in neural development, PI3K-AKT/mTOR signaling or stress-immune regulation [22,23,24,25,26]. In silico knockout of each induced significant differential expression (Supplemental Tables S10 and S11; Supplemental Figure S3), with recurrent responses involving mitochondrial/redox metabolism, cytoskeletal remodeling, chromatin/epigenetic regulation and immune-related signaling. Notably, FKBP5 knockout also affected CX3CR1, a canonical microglial marker and immune receptor. These results provide independent computational support that the model can prioritize biologically plausible candidate regulators not directly recorded in the training graph, but they should not be interpreted as direct experimental evidence of causal regulation.

Overall, the knowledge graph embedding organizes existing evidence and enables hypothesis-driven prioritization of potential cell-type-specific regulators. Predictions should be interpreted as candidate hypotheses, not causal conclusions. Having evaluated its link-prediction performance, we next compared our knowledge graph with existing resources.

### 2.6. Systematic Feature Comparison with Representative Resources

To define the distinctive characteristics of our knowledge graph for organizing neural cell regulatory knowledge, we selected five representative resources and methods and compared them across six functional dimensions, predefined according to the functional requirements of this study: cell-type focus, literature evidence traceability, regulatory relation modeling, direction/effect annotation, single-cell molecular fingerprint integration and knowledge graph reasoning. The included resources were CellMarker/PanglaoDB, PuMA, PubTator 3.0, MEDMKG and BioPathNet [6,7,13,17,27,28]. The comparison results are summarized in Table 1. Throughout the manuscript, we describe Table 1 as a feature-level comparison or qualitative feature summary, rather than a scoring system, rating or ranking.

Among the compared resources, only our study built a hierarchical neural cell type taxonomy anchored to the Cell Ontology. Our graph explicitly encodes REGULATES edges between perturbagens and cell types with annotated effect direction and perturbation type attributes, stores literature-derived evidence records, and integrates quantitative single-cell molecular fingerprints. Additionally, it supports knowledge graph embedding for predicting unobserved regulatory relationships.

To compare resources on a common basis, we selected six functional dimensions that collectively span cell-type representation, evidence provenance, regulatory semantics and computational utility: cell-type focus, literature evidence traceability, regulatory relation modeling, direction/effect annotation, single-cell molecular fingerprint integration and knowledge graph reasoning. Each resource was assessed against its official documentation and representative queries, and it was categorized as ✓ (full support: the feature is explicitly implemented and documented), ∆ (partial or indirect support: the feature is present but limited in scope, granularity or accessibility) or – (no explicit support). We note three limitations of this scheme: it captures feature presence rather than quantitative performance; the boundary between ✓ and ∆ involves subjective judgment; and feature assessment is conducted by a single rater without independent multi-rater replication. The comparison is therefore intended to highlight structural complementarity across resource classes rather than to rank resources within any single dimension.

By contrast, the existing marker databases (CellMarker, PanglaoDB) provide only static gene–cell associations without causal direction. Text-mining systems (PuMA, PubTator 3.0) lack directional semantics or cell-type specificity. General biomedical knowledge graphs (MEDMKG) focus on clinical tasks rather than cell-centric regulation. BioPathNet supports link prediction but lacks cell type hierarchies and molecular fingerprints. This combination of features positions our resource as a computable foundation for hypothesis generation and regulatory knowledge organization in neuroscience.

## 3. Discussion

This study presents a multimodal knowledge graph framework anchored on neural cell types. The framework integrates a hierarchical cell taxonomy, single-cell transcriptome-derived molecular fingerprints, literature-extracted regulatory evidence and gene–gene functional interaction information into a unified graph structure. Unlike general biomedical knowledge graphs that are primarily organized around genes, diseases or drugs [5,6,7], this resource uses neural cell types as the organizing coordinate system, allowing regulatory evidence to be queried and reasoned over at multiple levels of cellular granularity.

A key methodological contribution is the use of a hierarchical cell-type framework to mitigate the granularity mismatch between literature-derived evidence and single-cell transcriptomic data. Descriptions of cell types in the neuroscience literature often use inconsistent nomenclature and variable resolution [2,9,10], whereas transcriptome-based classifications define cell identity at molecular resolution. By retaining fine-grained literature anchors while assigning molecular fingerprints only at transcriptomically resolvable levels, the framework avoids over-interpreting unsupported molecular specificity and provides a practical route for cross-study alignment.

The knowledge graph embedding results suggest that cellular molecular identity and literature-derived regulatory evidence can be jointly represented in a shared embedding space. By modeling cell–factor regulatory relations, cell–gene marker relations and gene–gene interactions together, the model prioritizes candidate regulators and marker genes in a cell-type-specific manner. The microglia case study illustrates how such predictions can recover biologically plausible inflammatory and immune-related candidates while remaining explicitly framed as hypotheses rather than direct causal evidence.

Several limitations should be acknowledged. First, regulatory relations were extracted primarily from PubMed abstracts, which may omit full-text experimental details such as dosage, time scale, experimental system and negative results. Second, the framework does not yet incorporate perturbation-induced transcriptomic datasets such as Perturb-seq or CRISPR screens, which would provide direct molecular response evidence [3,4]. Third, the current hierarchy is a curated core taxonomy rather than an exhaustive representation of all neural cell states. Future work should extend the graph with full-text evidence extraction, perturbational single-cell datasets, spatial and electrophysiological modalities, and more systematic entity normalization.

The use of an LLM for literature extraction also introduces a measurable source of uncertainty. LLM-based pipelines can introduce several specific risks, including hallucinated relations, terminology normalization bias, abstract-level evidence gaps and propagation of extraction errors into downstream graph reasoning. Our expert validation provides a quantitative estimate of these risks: across 331 triples spanning six cell types with varying literature coverage, overall extraction accuracy was 96.4%, with inter-annotator agreement of 99.7% (Cohen’s κ=0.955). Accuracy was not uniformly distributed across cell types: well-studied populations such as microglia (98.0%), oligodendrocyte precursor cells (100%) and dorsal raphe serotonergic neurons (100%) reached near-perfect accuracy, whereas dopaminergic neurons (92.0%) and chandelier cells (91.3%) showed moderately lower accuracy. Notably, within this validation set, we did not observe a clear systematic decrease in accuracy for rare cell types. However, the small number of pia-cell records warrants cautious interpretation, suggesting that both literature heterogeneity and evidence complexity may contribute to residual errors. Qualitative analysis of the five relation-logic errors identified three recurrent failure patterns—direction reversals in loss-of-function contexts, causal-polarity confusion in protective perturbations, and misclassification of non-regulatory events as REGULATES edges—indicating that residual errors concentrate in semantically complex regulatory statements rather than in entity recognition or cell-type mapping. To mitigate error propagation, all the identified errors were corrected during manual curation, and triples with confidence scores below 8 were excluded from downstream analysis. Future work should incorporate full-text extraction, structured ontology grounding and cross-model consistency checks to further constrain LLM hallucination and terminology drift.

Another limitation is the uneven recovery of canonical markers across cell types. Although the overall CellMarker recovery rate was 57.7%, several fine-grained or anatomically specialized cell types showed low or no recovery under the strict Top 50 criterion. This may reflect differences between CellMarker labels and atlas-derived annotations, the one-versus-rest differential expression strategy, and mismatched granularity between literature-defined cell types and transcriptome-based states. Since MARKER_OF is only one component of the KGE input, alongside REGULATES, INTERACTS_WITH and hierarchical relationships, low recovery in some cell types does not invalidate the overall graph. However, KGE predictions for these cell types should be interpreted with caution and require further validation.

The feature-level comparison in Table 1 also has limitations. Although the criteria were predefined according to the functional requirements of this study, the ✓/∆/– symbols remain qualitative support categories and may involve interpretation for resources with partially overlapping functions. We therefore clarified the symbol definitions, avoided describing the table as a scoring or ranking system, and restricted the comparison to observable functions described in the original resources. Standardized quantitative benchmarks for neural-cell-specific knowledge graphs are not yet established, and the compared resources differ in scope, data model and intended use case, making direct head-to-head performance comparison infeasible at present. Future work should develop community benchmarks to enable quantitative cross-resource evaluation as such standards emerge.

In summary, this study provides an extensible framework for integrating and reasoning over cell-type-specific regulatory knowledge in neuroscience. By unifying hierarchical cell semantics, molecular fingerprints, literature evidence and gene–gene functional interactions, the framework enables candidate regulator prioritization, marker gene retrieval and prediction of unobserved regulatory relations.

## 4. Materials and Methods

### 4.1. Construction of the Neural Cell Hierarchy

The knowledge graph required a unified semantic backbone for integrating transcriptomic data, literature evidence and gene interaction information. We used the Cell Ontology as the primary reference framework [8,9] and refined it using cell-type annotations from published human brain single-cell atlases, including early developmental and adult human brain datasets [11,12]. The goal was not to reproduce the full Cell Ontology, but to build a compact, traceable and interpretable neural cell hierarchy suitable for graph construction and downstream link prediction.

Candidate cell types were collected from three sources: Cell Ontology terms, atlas annotations and literature-derived neural cell terms. To improve reproducibility, the expert-guided pruning procedure was formalized into a rule-based inclusion and exclusion workflow. Candidate terms were first normalized by Cell Ontology identifier mapping, synonym merging and alias retention. Terms with identical ontology identifiers, exact synonymy, spelling variants, abbreviations or dataset-specific labels referring to the same biological population were merged into a single canonical node, while the original names were retained as aliases for literature mapping.

Candidate terms were included in the core hierarchy if they satisfied at least one of the following criteria: direct or close mapping to a nervous-system-relevant Cell Ontology term; occurrence as an annotated cell type or subtype in at least one reference single-cell atlas; occurrence as a target cell type in retained literature-derived regulatory evidence with an unambiguous parent assignment; or biological interpretability as a neural, glial, progenitor, vascular, meningeal or other nervous-system-associated cell population defined by lineage, function, anatomical origin or established nomenclature.

Candidate terms were merged, mapped to an upper-level node or excluded according to predefined operational rules. A term was considered redundant if it represented a synonym, abbreviation, spelling variant or dataset-specific label of an already included node. A term was considered overly broad if it did not provide additional biological specificity beyond an existing upper-level category. A term was considered ambiguous if it could be assigned to more than one parent lineage or functional class and the available ontology, atlas or literature context was insufficient for disambiguation. A term was considered unsupported if it lacked support from Cell Ontology, reference atlas annotations or retained literature evidence.

The remaining terms were organized into three levels. Level 1 contains four major classes: Neuron, Glial cell, Progenitor and Non-neural cell of the nervous system. Level 2 contains 30 functional cell types defined by functional characteristics, lineage relationships or anatomical origin. Level 3 contains 45 fine-grained subtypes that capture higher-resolution transcriptomic or literature-specific distinctions. Level 3 nodes are connected to Level 2 nodes through BELONGS_TO relationships, and Level 2 nodes are connected to Level 1 nodes through PART_OF relationships.

Taxonomic inclusion and transcriptomic molecular anchoring were annotated separately. A node was assigned a direct molecular anchor only when it appeared as a resolvable annotation category in the reference single-cell atlases and yielded marker genes under the differential-expression criteria described in Section 4.2. Nodes supported by ontology or literature evidence but not independently resolved in the transcriptomic atlases were retained as semantic hierarchy nodes. Their molecular context, when applicable, was propagated from the nearest transcriptomically supported parent node or summarized from directly anchored descendant nodes, depending on the hierarchy position. These propagated or summarized molecular features were used only as hierarchical context and were not interpreted as independent subtype-specific marker evidence.

Each cell type node was assigned a textual definition and a source identifier where available. Cell Ontology identifiers were retained when a direct or close mapping existed. For curated terms without a direct Cell Ontology identifier, definitions and evidence sources were manually recorded. When identical or similar canonical names occurred at different levels, nodes were disambiguated by their unique node identifiers, hierarchy levels and parent-node assignments.

The rule-based inclusion and exclusion workflow is summarized in Supplemental Figure S4. The final included hierarchy, including canonical names, parent nodes, available Cell Ontology identifiers, evidence sources and molecular-anchor status, is provided in Supplemental Table S1. Because this study aimed to construct a compact core hierarchy rather than an exhaustive reconstruction of all candidate ontology and literature terms, we report the complete list of included nodes and define exclusion criteria at the rule level.

### 4.2. Single-Cell Transcriptomic Marker Gene Identification and Validation

Two published human brain single-cell RNA-seq datasets were integrated as a steady-state molecular reference: an adult human brain atlas containing 2.37 million cells and an early developmental atlas containing 1.78 million cells. Together, these datasets covered 4.16 million cells and approximately 59,000 genes. Count matrices were merged across cell types and normalized in Seurat using LogNormalize with a scale factor of 10,000 [29].

Differential expression analysis was performed using Seurat FindAllMarkers in a one-versus-rest design with the Wilcoxon rank-sum test [29]. Only positive markers were tested and retained using the following parameters: min.pct = 0.25, logfc.threshold = 0.25 and only.pos = TRUE. Differentially expressed genes were filtered by adjusted p<0.05 and ranked within each cell type by average log_2_ fold change. Gene identifiers were converted from Ensembl identifiers to gene symbols where possible, and genes annotated as protein-coding in the gene biotype annotation were retained for the final marker set. For each cell type, the Top 50 protein-coding genes ranked by average log_2_ fold change were selected as candidate molecular fingerprints. The Top 50 cutoff follows the default reporting convention of Seurat FindAllMarkers for cluster markers [29] and was chosen to balance coverage of cell-type-defining genes against visual readability in downstream panels; it operated downstream of the statistical filters (min.pct, logfc.threshold, adjusted *p*) and served as a presentation threshold rather than a statistical cutoff. This produced 1550 cell type–marker gene records covering 31 transcriptomically supported cell types and 1318 non-redundant protein-coding genes.

The candidate marker sets were externally evaluated against CellMarker 2.0 [13]. Across 20 cell types and 97 classic markers, 56 appeared in the corresponding Top 50 candidate lists, yielding an overall hit rate of 57.7%. Cell-type-level recovery rates, recovery categories, recovered markers and missing CellMarker-listed markers were summarized in Supplemental Table S3 to distinguish well-recovered, partially recovered and low-recovery cell types. For graph construction, marker genes were represented as Gene nodes and connected to cell type nodes through MARKER_OF relationships.

Because transcriptomic annotations did not always resolve the same granularity as the literature-based hierarchy, molecular anchors were assigned only at levels supported by the transcriptome data. For example, when transcriptomic annotations grouped hippocampal CA1–CA3 pyramidal neurons together, molecular fingerprints were assigned to the upper Pyramidal neuron node rather than to unsupported fine-grained subtypes. Hierarchical relationships were then used to share molecular context with child nodes while avoiding unsupported subtype-specific claims.

### 4.3. Llm-Driven Regulatory Relation Extraction and Quality Control

PubMed abstracts were mined to extract cell-type-specific regulatory evidence. Literature retrieval used cell type names, synonyms and hierarchy-aware query terms to collect candidate abstracts across Level 1, Level 2 and Level 3 cell types. For each target cell type, the primary name and curated aliases were combined as quoted PubMed search terms using OR logic. PubMed records were retrieved with NCBI Entrez, ranked by relevance and processed in batches of five abstracts, with cross-cell-type PMID deduplication before LLM screening.

LLM inference used the OpenAI-compatible chat-completions API provided by CSTCloud Uni-API, with the S1-Base-Ultra model for both screening and extraction. Calls were run asynchronously with a maximum concurrency of three, up to three re-tries per request, a 60 s request timeout and exponential back-off. Decoding was deterministic, with temperature set to 0.0, streaming disabled and maximum output lengths of 500 tokens for screening and 4000 tokens for extraction. Prompt templates are provided in the release files.

The LLM workflow had two stages [30,31]. First, candidate abstracts were screened using a biomedical screener prompt requiring explicit evidence for the target neural cell term, a perturbing factor and a molecular or cellular response in the same cell context. The screening output was restricted to JSON containing the PMID and a Boolean relevance field. Second, relevant abstracts were processed with a structured extraction prompt requiring a JSON list of perturbation–effect records. Each record contained PMID, factor name, factor type at two controlled-vocabulary levels, perturbation action, target cell type, regulatory effect, effect direction, evidence sentence and confidence score. The extraction prompt required one perturbation and one downstream effect per record, exact evidence from the abstract, no unsupported inference and an empty list when no valid perturbation–effect pair was present.

Extracted cell type terms were mapped to the curated hierarchy. Fine-grained terms were mapped to Level 3 when possible, whereas broader or ambiguous descriptions were conservatively mapped to Level 2 or Level 1. Quality control combined automated JSON parsing and schema checks, removal of records with missing essential fields or invalid effect direction, LLM-assisted consistency assessment and manual curation. Records with unsupported cell context or inconsistent target mapping were removed or corrected. The final evidence set contained 25,812 curated regulatory evidence records, including 544 Level 1 records, 6547 Level 2 records and 18,721 Level 3 records. During Neo4j import, records with identical factor–cell–PMID–effect combinations were merged, yielding 25,202 REGULATES relationships in the complete graph: 527 Level 1, 6404 Level 2 and 18,271 Level 3 targets.

Extraction accuracy was evaluated using 331 sampled records spanning six cell types: 100 dopaminergic neurons, 100 microglia, 50 oligodendrocyte precursor cells, 50 dorsal raphe serotonergic neurons, 23 chandelier cells and 8 pia cells. Two domain experts independently evaluated the records against a predefined gold standard. The overall extraction accuracy was 96.4% (319/331), with 92.0% for dopaminergic neurons, 98.0% for microglia, 100.0% for oligodendrocyte precursor cells, 100.0% for dorsal raphe serotonergic neurons, 91.3% for chandelier cells and 100.0% for pia cells. The dimension-specific accuracies were 99.4% for entity recognition, 98.8% for cell type mapping and 98.5% for relation logic.

### 4.4. Multi-Source Knowledge Graph Integration and Neo4j Implementation

The graph was implemented in Neo4j as a property graph. The core node labels included Level1, Level2, Level3, Gene, Factor and Paper. The core relationship types included BELONGS_TO, PART_OF, MARKER_OF, REGULATES, MENTIONS and INTERACTS_WITH. The complete Neo4j graph contains 18,609 nodes and 41,532 directed relationships. The node counts are 9782 Factor nodes, 7463 Paper nodes, 1285 Gene nodes, 45 Level3 nodes, 30 Level2 nodes and 4 Level1 nodes. The relationship counts are 25,202 REGULATES, 12,349 MENTIONS, 2406 INTERACTS_WITH, 1500 MARKER_OF, 45 BELONGS_TO and 30 PART_OF relationships.

The hierarchy backbone was imported first. Marker genes were then imported as Gene nodes and connected to their target cell type nodes with MARKER_OF relationships. Literature-derived regulatory evidence was imported by mapping each target cell type to an existing hierarchy node, creating or reusing Factor and Paper nodes, connecting Paper nodes to Factor nodes through MENTIONS relationships and connecting Factor nodes to target cell nodes through REGULATES relationships. REGULATES relationships retained evidence-level attributes including PMID, effect, effect direction, perturbation action, evidence sentence and confidence score.

Gene–gene functional interactions were obtained from STRING using the marker gene set as the query space [32]. Only interactions for which both genes were present in the graph were retained. Self-loops and duplicate unordered gene pairs were removed. The complete Neo4j graph retained 2406 INTERACTS_WITH relationships; for KGE training, the STRING interaction set was represented as 4940 directed INTERACTS_WITH triples after direction expansion and preprocessing.

The graph quality control used Cypher queries to verify node and relationship counts, hierarchy path completeness, marker mapping, evidence traceability and gene interaction validity. For the KGE training, Paper nodes and MENTIONS relationships were excluded to avoid bias from document frequency and repeated evidence. The exported non-paper subgraph retained BELONGS_TO, PART_OF, MARKER_OF, REGULATES and INTERACTS_WITH relationships.

### 4.5. Knowledge Graph Embedding and Link Prediction

The KGE input was constructed by exporting a deduplicated head–relation–tail table from the Neo4j graph. The complete Neo4j graph contained 41,532 relationships, including 12,349 MENTIONS relationships linking Paper nodes to Factor nodes for evidence traceability. For KGE training, Paper nodes and their MENTIONS relationships were excluded to avoid bias from document frequency and repeated evidence, retaining only entity-to-entity relationships. The initial non-paper export contained 29,183 triples (25,202 REGULATES, 2406 INTERACTS_WITH, 1500 MARKER_OF, 45 BELONGS_TO and 30 PART_OF) [33].

To enhance marker gene coverage for cell-type-specific prediction tasks, we supplemented the graph with 810 additional MARKER_OF relationships from the CellMarker 2.0 database, yielding 2310 MARKER_OF triples in total. The INTERACTS_WITH relationships were expanded from 2406 to 4940 triples by including bidirectional edges for symmetric protein–protein interactions from STRING. REGULATES relationships were deduplicated by merging records with identical head–tail pairs but different PMID sources, reducing the count from 25,202 to 12,494 unique regulatory relationships while preserving all distinct Factor–Cell pairs.

The final KGE dataset contained 19,819 triples over 10,660 entities and five relation types: 12,494 REGULATES, 2310 MARKER_OF, 4940 INTERACTS_WITH, 45 BELONGS_TO and 30 PART_OF triples. The entity prefixes included 9782 Factor entities, 799 Gene entities, 45 Level3 entities, 30 Level2 entities and 4 Level1 entities.

A relation-specific transductive-safe splitting strategy was used. BELONGS_TO and PART_OF relationships define the taxonomy backbone and were retained in the training set. REGULATES triples were split into training, validation and test sets at an approximate 8:1:1 ratio and were used for early stopping and primary link prediction evaluation. MARKER_OF and INTERACTS_WITH relations were split into training and held-out test sets for auxiliary evaluation. Any validation or test triple containing an entity absent from the training set was moved back to the training set. For INTERACTS_WITH, unordered gene pairs were split as pairs to prevent the two directions of the same interaction from being assigned to different splits.

In the final seed 42 split, the training set contained 17,822 triples. The REGULATES validation and test sets contained 383 and 378 triples, respectively. The MARKER_OF and INTERACTS_WITH held-out test sets contained 320 and 916 triples, respectively. No unseen entities remained in the validation or test sets, and no triples overlapped across splits. To verify that the 8:1:1 partition did not bias the reported REGULATES performance, we conducted a split-level robustness sweep using RotatE with five independent 8:1:1 transductive splits (split seeds 1–5, training seed fixed at 42). Across the five splits, the mean filtered MRR was 0.369 ± 0.028 (CV = 7.6%), and the originally reported result (MRR = 0.373) fell within the observed range and close to the mean (Supplemental Tables S8 and S9).

The KGE models were trained using PyKEEN [34]. RotatE was selected as the final model because it can represent symmetric, antisymmetric, inverse and compositional relation patterns and is suitable for directional regulatory relations [35]. The model parameters were: random seed 42, embedding dimension 256, maximum 500 epochs, batch size 2048, learning rate 0.001 and Adam optimizer. Training used stochastic local closed world assumption with a Bernoulli negative sampler, 128 negative samples per positive triple, and NSSALoss with margin 6.0 and adversarial temperature 1.0. Validation MRR on REGULATES was evaluated every 10 epochs and used for early stopping.

Model performance was evaluated using rank-based filtered evaluation [35,36]. For each test triple (h,r,t), head prediction fixed (r,t) and ranked candidate head entities over the full entity vocabulary, whereas tail prediction fixed (h,r) and ranked candidate tail entities over the full entity vocabulary. In the filtered setting, known true triples from the training, validation or test sets were removed from negative candidates. The metrics included mean reciprocal rank, Hits@1, Hits@3 and Hits@10. Because REGULATES was defined as Factor–REGULATES–Cell, predicting upstream regulators for a given cell type corresponded to head prediction. For cell-type-specific hypothesis generation, we additionally performed type-restricted scoring by ranking Factor entities for a fixed target cell type.

### 4.6. In Silico Knockout Validation

To evaluate whether KGE-ranked microglia regulator candidates could induce microglia-relevant molecular responses, we performed in silico knockout analysis following the scTenifoldKnk framework, an efficient virtual knockout approach for gene function prediction based on perturbation of single-cell gene regulatory networks that was originally described in Patterns in 2022 [37]. The expression input was the Microglia subset from the Comprehensive cell atlas of the first-trimester developing human brain [12]. FMR1, PTEN and FKBP5 were selected because they ranked highly in the Microglia REGULATES head-prediction task, did not have a direct REGULATES edge to Microglia in the training graph, and had interpretable neural, immune or stress-related biological context.

The Microglia expression matrix was stored in the Matrix Market format together with matched gene-name and cell-barcode files. For each candidate gene, wild-type and knockout gene regulatory networks were constructed from the same microglia single-cell expression matrix. The implementation followed the main steps of scTenifoldKnk: quality control of the expression matrix, construction of multiple single-cell gene regulatory networks, tensor decomposition to obtain a consensus wild-type network, virtual knockout by setting the target gene connections to zero, manifold alignment between wild-type and perturbed networks, and differential regulation analysis based on the distance between aligned gene embeddings. The expression matrix was downsampled to 500 cells for input loading, and each network construction sampled 300 cells. Wild-type networks were constructed 10 times. Networks were built using three principal components, and low-confidence network edges were filtered before tensor decomposition.

Genes with large standardized distance shifts between the wild-type and knockout manifolds were treated as differentially regulated genes. Nominal *p* values were computed from the distance-derived fold-change statistic and adjusted for multiple testing to obtain adjusted *p* values. The full gene-level ranking tables and significant differentially regulated gene lists are provided in the virtual knockout source data and summarized in Supplemental Tables S10 and S11. These analyses were used only as computational support for prioritization and were not interpreted as direct causal validation.

### 4.7. Statistical Analysis and Reproducibility

No statistical method was used to predetermine sample size. No data were excluded during analysis. No randomization or blinding was applied during data curation, model training or evaluation. The KGE models were trained and evaluated on fixed training, validation and test splits. The performance metrics were computed on held-out test sets. The release files for reproducing the major analyses include the cell hierarchy, marker gene tables, PubMed PMID lists, literature-derived regulatory evidence, STRING PPI tables, KGE train/validation/test splits, model evaluation results, LLM prompt templates and the 331 expert-validated records.

## Figures and Tables

**Table 1:** Feature-level comparison between this study and representative related works.

Work	CT- Centered	Evidence Traceable	Regulatory Knowledge	scRNA-seq Fingerprints	KG Inference	Neural-Cell Specific
CellMarker 2.0/PanglaoDB	✓	∆	–	∆	–	∆
PuMA	✓	✓	∆	–	–	∆
PubTator 3.0	∆	✓	✓	–	–	–
MEDMKG	–	∆	✓	–	✓	–
BioPathNet	–	–	✓	–	✓	–
**This study**	✓	✓	✓	✓	✓	✓

*Notes:* ✓, explicitly implemented or directly supported; ∆, partially, indirectly or context-dependently supported; –, not explicitly provided or described. The symbols denote support categories for a qualitative feature-level comparison and should not be interpreted as a quantitative score, rating, ranking or superiority assessment. Assessment was performed against official documentation of each resource; see main text for methodology and limitations. Regulatory knowledge includes regulatory relationship modeling and direction/effect information when available. CT-centered, cell-type-centered representation; KG, knowledge graph.

## Data Availability

All source code, data files, and Supplementary Materials are publicly available at https://github.com/SiatBioInf/NeuroCellKG (accessed on 22 July 2026). The repository includes knowledge graph construction pipelines, KGE model training scripts, LLM extraction prompt templates, expert validation samples, and all data files described in the manuscript. Single-cell transcriptomic datasets were obtained from published studies (Braun et al., Science 2023; Siletti et al., Science 2023) [11,12]. Cell Ontology definitions are available at http://www.obofoundry.org/ontology/cl.html (accessed on 22 July 2026). STRING protein–protein interaction data are available at https://string-db.org/ (accessed on 22 July 2026).

## Supplementary Materials

The following supporting information can be downloaded at: https://www.mdpi.com/article/10.3390/ijms27156842/s1.

## References

[1] Fernandez-Moya S.M., Ganesh A.J., Plass M. (2023). Neural cell diversity in the light of single-cell transcriptomics. Transcription.

[2] Mukamel E.A., Ngai J. (2019). Perspectives on defining cell types in the brain. Curr. Opin. Neurobiol..

[3] Bonev B., Castelo-Branco G., Chen F., Codeluppi S., Corces M.R., Fan J., Heiman M., Harris K., Inoue F., Kellis M. (2024). Opportunities and challenges of single-cell and spatially resolved genomics methods for neuroscience discovery. Nat. Neurosci..

[4] Adameyko I., Bakken T., Bhaduri A., Chhatbar C., Filbin M.G., Gate D., Hochgerner H., Kim C.N., Krull J., La Manno G. (2024). Applying single-cell and single-nucleus genomics to studies of cellular heterogeneity and cell fate transitions in the nervous system. Nat. Neurosci..

[5] Cui H., Lu J., Xu R., Wang S., Ma W., Yu Y., Yu S., Kan X., Ling C., Zhao L. (2025). A review on knowledge graphs for healthcare: Resources, applications, and promises. J. Biomed. Inform..

[6] Wang X., Zhong Y., Zhang L., Dai L., Wang T., Ma F. (2025). MEDMKG: Benchmarking Medical Knowledge Exploitation with Multimodal Knowledge Graph. arXiv.

[7] Hu E.Y., Oleshko S., Firmani S., Cheng H., Zhu Z., Ulmer M., Arnold M., Colome-Tatche M., Tang J., Xhonneux S. (2026). Enhancing link prediction in biomedical knowledge graphs with BioPathNet. Nat. Biomed. Eng..

[8] Tan S.Z.K., Puig-Barbe A., Goutte-Gattat D., Eastwood C., Aevermann B., Avola A., Balhoff J.P., Bayindir I.U., Belfiore J., Caron A.R. (2026). The Cell Ontology in the age of single-cell omics. Sci. Data.

[9] Tan S.Z.K., Kir H., Aevermann B.D., Gillespie T., Harris N., Hawrylycz M.J., Jorstad N.L., Lein E.S., Matentzoglu N., Miller J.A. (2023). Brain Data Standards—A method for building data-driven cell-type ontologies. Sci. Data.

[10] Miller J.A., Gouwens N.W., Tasic B., Collman F., van Velthoven C.T., Bakken T.E., Hawrylycz M.J., Zeng H., Lein E.S., Bernard A. (2020). Common cell type nomenclature for the mammalian brain. eLife.

[11] Siletti K., Hodge R., Mossi Albiach A., Lee K.W., Ding S.L., Hu L., Lonnerberg P., Bakken T., Casper T., Clark M. (2023). Transcriptomic diversity of cell types across the adult human brain. Science.

[12] Braun E., Danan-Gotthold M., Borm L.E., Lee K.W., Vinsland E., Lonnerberg P., Hu L., Li X., He X., Andrusivova Z. (2023). Comprehensive cell atlas of the first-trimester developing human brain. Science.

[13] Hu C., Li T., Xu Y., Zhang X., Li F., Bai J., Chen J., Jiang W., Yang K., Ou Q. (2023). CellMarker 2.0: An updated database of manually curated cell markers in human/mouse and web tools based on scRNA-seq data. Nucleic Acids Res..

[14] Owens G.K., Kumar M.S., Wamhoff B.R. (2004). Molecular Regulation of Vascular Smooth Muscle Cell Differentiation in Development and Disease. Physiol. Rev..

[15] Aird W. (2007). Phenotypic heterogeneity of the endothelium: I. Structure, function, and mechanisms. Circ. Res..

[16] Nishiyama A., Komitova M., Suzuki R., Zhu X. (2009). Polydendrocytes (NG2 cells): Multifunctional cells with lineage plasticity. Nat. Rev. Neurosci..

[17] Bickmann L., Sandmann S., Walter C., Varghese J. (2025). PuMA: PubMed gene/cell type-relation Atlas. BMC Bioinform..

[18] Yu J., Zhu H., Taheri S., Mondy W., Bonilha L., Magwood G.S., Lackland D., Adams R.J., Kindy M.S. (2019). Serum Amyloid A-Mediated Inflammasome Activation of Microglial Cells in Cerebral Ischemia. J. Neurosci..

[19] Garate I., Garcia-Bueno B., Madrigal J.L.M., Caso J.R., Alou L., Gomez-Lus M.L., Leza J.C. (2014). Toll-like 4 receptor inhibitor TAK-242 decreases neuroinflammation in rat brain frontal cortex after stress. J. Neuroinflamm..

[20] Butovsky O., Jedrychowski M.P., Moore C.S., Cialic R., Lanser A.J., Gabriely G., Koeglsperger T., Dake B., Wu P.M., Doykan C.E. (2014). Identification of a unique TGF-beta-dependent molecular and functional signature in microglia. Nat. Neurosci..

[21] Zhang Q., Tao W., Wang J., Qian M., Zhou M., Gao L. (2025). The OLR1/NF-kappaB feedback loop exacerbates HIV-1 Tat-induced microglial inflammatory response and neuronal apoptosis. J. Neurovirol..

[22] Parrott J.M., Oster T., Lee H.Y. (2021). Altered Inflammatory Response in FMRP-deficient Microglia. iScience.

[23] Zhou X., Wei J., Li L., Shu Z., You L., Liu Y., Zhao R., Yao J., Wang J., Luo M. (2022). Microglial Pten Safeguards Postnatal Integrity of the Cortex and Sociability. Front. Immunol..

[24] Li Y., Qiu Y., Yang Y., Wei Y., Peng H., Zeng L., Li P., Bi R., Hu B. (2026). Microglial Fkbp5 Impairs Post-Stroke Vascular Integrity and Regeneration by Promoting Yap1-Mediated Glycolysis and Oxidative Phosphorylation. Adv. Sci..

[25] Qiu B., Xu Y., Wang J., Liu M., Dou L., Deng R., Wang C., Williams K.E., Stewart R.B., Xie Z. (2019). Loss of FKBP5 Affects Neuron Synaptic Plasticity: An Electrophysiology Insight. Neuroscience.

[26] Lauro C., Chece G., Monaco L., Antonangeli F., Peruzzi G., Rinaldo S., Paone A., Cutruzzolà F., Limatola C. (2019). Fractalkine Modulates Microglia Metabolism in Brain Ischemia. Front. Cell. Neurosci..

[27] Franzen O., Gan L.M., Bjorkegren J.L.M. (2019). PanglaoDB: A web server for exploration of mouse and human single-cell RNA sequencing data. Database.

[28] Wei C.H., Allot A., Lai P.T., Leaman R., Tian S., Luo L., Jin Q., Wang Z., Chen Q., Lu Z. (2024). PubTator 3.0: An AI-powered literature resource for unlocking biomedical knowledge. Nucleic Acids Res..

[29] Hao Y., Stuart T., Kowalski M.H., Choudhary S., Hoffman P., Hartman A., Srivastava A., Molla G., Madad S., Fernandez-Granda C. (2024). Dictionary learning for integrative, multimodal and scalable single-cell analysis. Nat. Biotechnol..

[30] Fornasiere R., Brunello N., Scotti V., Carman M., Abbas M., Freihat A.A. (2024). Medical Information Extraction with Large Language Models. Proceedings of the 7th International Conference on Natural Language and Speech Processing (ICNLSP 2024).

[31] Johnson J.A., Bergman D.R., Rocha H.L., Zhou D.L., Cramer E., Mclean I.C., Dance Y.W., Booth M., Nicholas Z., Lopez-Vidal T. (2025). Human interpretable grammar encodes multicellular systems biology models to democratize virtual cell laboratories. Cell.

[32] Szklarczyk D., Kirsch R., Koutrouli M., Nastou K., Mehryary F., Hachilif R., Gable A.L., Fang T., Doncheva N.T., Pyysalo S. (2023). The STRING database in 2023: Protein–protein association networks and functional enrichment analyses for any sequenced genome of interest. Nucleic Acids Res..

[33] Wang Q., Mao Z., Wang B., Guo L. (2017). Knowledge Graph Embedding: A Survey of Approaches and Applications. IEEE Trans. Knowl. Data Eng..

[34] Ali M., Berrendorf M., Hoyt C.T., Vermue L., Sharifzadeh S., Tresp V., Lehmann J. (2021). PyKEEN 1.0: A Python Library for Training and Evaluating Knowledge Graph Embeddings. J. Mach. Learn. Res..

[35] Sun Z., Deng Z.H., Nie J.Y., Tang J. RotatE: Knowledge Graph Embedding by Relational Rotation in Complex Space. Proceedings of the International Conference on Learning Representations.

[36] Bordes A., Usunier N., Garcia-Duran A., Weston J., Yakhnenko O. (2013). Translating Embeddings for Modeling Multi-relational Data. Proceedings of the Advances in Neural Information Processing Systems.

[37] Osorio D., Zhong Y., Li G., Xu Q., Yang Y., Tian Y., Chapkin R.S., Huang J.Z., Cai J.J. (2022). scTenifoldKnk: An efficient virtual knockout tool for gene function predictions via single-cell gene regulatory network perturbation. Patterns.

