# Supplementary material for: A Hierarchical Multimodal Knowledge Graph for Neural Cell-Type-Specific Regulation Integrating Single-Cell Transcriptomics and Literature Evidence
Source: Int J Mol Sci. 2026 Jul 30;27(15):6842. doi: 10.3390/ijms27156842 (PMC13466513; doi:10.3390/ijms27156842)
Supplement: Supplementary file 1 [file ijms-27-06842-s001.zip › ijms-4387846-supplementary.pdf]

Supplementary materials

Hierarchical multimodal knowledge graph for neural cell-type-specific regulation

Supplementary materials are organized in the same order as the main manuscript: cell hierarchy, single-cell marker validation, LLM extraction validation, graph/KGE statistics and in silico knockout validation. Large source-data files, code and prompt templates are provided in the public release package to be deposited with the manuscript.

Supplementary Table 1. Final 79-node neural cell hierarchy (split into 1a and 1b for readability)

This table lists all cell-type nodes included in the curated neural cell hierarchy. Each row represents one hierarchy node and includes its unique node identifier, canonical name, hierarchy level, immediate parent node, biological definition, supporting evidence source, available Cell Ontology identifier and molecular-anchor status. The molecular_anchor_status field has three possible values. direct indicates that the node appeared as a resolvable annotation category in the reference single-cell atlas and had marker genes identified under the stated differential-expression criteria. propagated indicates that the node did not have an independent marker profile, but molecular context was propagated from the nearest transcriptomically supported parent or child node. semantic-only indicates that the node was retained as an ontology- or literature-supported semantic anchor, but no molecular marker information was assigned to it. When a canonical cell-type name occurred at both Level 2 and Level 3, the two entries were treated as distinct hierarchy nodes and were disambiguated by node_id, hierarchy level and parent_node assignment. Molecular-anchor status was assigned according to the level at which the corresponding atlas annotation and marker set were represented in the graph. Parent-level nodes were annotated as propagated when their molecular context was derived from directly anchored child nodes rather than from an independent marker set. For nodes annotated as propagated, "scRNA-seq atlas" in the source field may indicate support from atlas-resolved descendant or parent nodes rather than an independent atlas annotation for that exact node. Nodes without a direct Cell Ontology identifier were marked as NA and retained when supported by reference atlas annotations, PubMed-derived regulatory evidence or manually curated literature definitions.

Supplementary Table 1a. Structural metadata for the 79-node neural cell hierarchy

Structural metadata for each hierarchy node: unique identifier, canonical name, hierarchy level, immediate parent, evidence source, Cell Ontology identifier and molecular-anchor status. Long textual definitions and per-node notes are provided in Supplementary Table 1b.

node_id	canonical_name	level	parent_node	source	Cell_Ontology_ID	molecular_anchor_status
node_id	canonical_name	level	parent_node	source	Cell_Ontology_ID	molecular_anchor_status
L1_Neuron	Neuron	Level 1	NA	Cell Ontology + literature evidence	CL:0000540	semantic-only
L1_Glial_cell	Glial cell	Level 1	NA	literature evidence	NA	semantic-only
L1_Non-neural_cell_of_nervous_system	Non-neural cell of nervous system	Level 1	NA	literature evidence	NA	semantic-only
L1_Progenitor	Progenitor	Level 1	NA	literature evidence	NA	semantic-only
L2_Excitatory_neuron	Excitatory neuron	Level 2	L1_Neuron	scRNA-seq atlas + literature evidence	NA	propagated
L2_Astrocyte	Astrocyte	Level 2	L1_Glial_cell	Cell Ontology + scRNA-seq atlas + literature evidence	CL:0000127	propagated

node_id	canonical_name	level	parent_node	source	Cell_Ontology_ID	molecular_anchor_status
L2_Macrophage	Macrophage	Level 2	L1_Glial_cell	Cell Ontology + literature evidence	CL:0000235	semantic-only
L2_Inhibitory_neuron	Inhibitory neuron	Level 2	L1_Neuron	scRNA-seq atlas + literature evidence	NA	propagated
L2_Interneuron	Interneuron	Level 2	L1_Neuron	Cell Ontology + scRNA-seq atlas + literature evidence	CL:0000099	propagated
L2_Epithelial_cell	Epithelial cell	Level 2	L1_Non-neural_cell_of_nervous_system	Cell Ontology + scRNA-seq atlas + literature evidence	CL:0000066	propagated
L2_Oligodendrocyte_precursor	Oligodendrocyte precursor	Level 2	L1_Progenitor	Cell Ontology + scRNA-seq atlas + literature evidence	CL:0002453	propagated
L2_Cortical_neuron	Cortical neuron	Level 2	L1_Neuron	Cell Ontology + scRNA-seq atlas + literature evidence	CL:0010012	propagated
L2_Medium_spiny_neuron	Medium spiny neuron	Level 2	L1_Neuron	Cell Ontology + scRNA-seq atlas + literature evidence	CL:1001474	propagated
L2_Endothelial_cell	Endothelial cell	Level 2	L1_Non-neural_cell_of_nervous_system	Cell Ontology + scRNA-seq atlas + literature evidence	CL:0000115	propagated
L2_Ependymal_cell	Ependymal cell	Level 2	L1_Non-neural_cell_of_nervous_system	Cell Ontology + scRNA-seq atlas + literature evidence	CL:0000065	propagated
L2_Fibroblast	Fibroblast	Level 2	L1_Non-neural_cell_of_nervous_system	Cell Ontology + scRNA-seq atlas + literature evidence	CL:0000057	propagated
L2_Granule_cell	Granule cell	Level 2	L1_Neuron	Cell Ontology + scRNA-seq atlas + literature evidence	CL:0000120	propagated
L2_Pyramidal_neuron	Pyramidal neuron	Level 2	L1_Neuron	Cell Ontology + scRNA-seq atlas + literature evidence	CL:0000598	direct
L2_Microglia	Microglia	Level 2	L1_Glial_cell	Cell Ontology + scRNA-seq atlas + literature evidence	CL:0000129	propagated
L2_Neural_crest_cell	Neural crest cell	Level 2	L1_Progenitor	Cell Ontology + scRNA-seq atlas + literature evidence	CL:0011012	propagated
L2_Neuroblast	Neuroblast	Level 2	L1_Progenitor	Cell Ontology + scRNA-seq atlas + literature evidence	CL:0000031	propagated
L2_Neuroplacodal_cell	Neuroplacodal cell	Level 2	L1_Progenitor	Cell Ontology + scRNA-seq atlas + literature evidence	CL:0000032	propagated
L2_Oligodendrocyte	Oligodendrocyte	Level 2	L1_Glial_cell	Cell Ontology + scRNA-seq atlas + literature evidence	CL:0000128	propagated
L2_Pericyte	Pericyte	Level 2	L1_Non-neural_cell_of_nervous_system	Cell Ontology + scRNA-seq atlas + literature evidence	CL:0000669	propagated
L2_Radial_glial_cell	Radial glial cell	Level 2	L1_Progenitor	Cell Ontology + scRNA-seq atlas + literature evidence	CL:0000681	propagated
L2_Smooth_muscle_cell	Smooth muscle cell	Level 2	L1_Non-neural_cell_of_nervous_system	Cell Ontology + scRNA-seq atlas + literature evidence	CL:0000192	propagated
L2_Dopaminergic_neuron	Dopaminergic neuron	Level 2	L1_Neuron	Cell Ontology + literature evidence	CL:0000700	semantic-only
L2_Cholinergic_neuron	Cholinergic neuron	Level 2	L1_Neuron	Cell Ontology + literature evidence	CL:0000108	semantic-only
L2_Serotonergic_neuron	Serotonergic neuron	Level 2	L1_Neuron	Cell Ontology + literature evidence	CL:0000850	semantic-only
L2_Sensory_neuron	Sensory neuron	Level 2	L1_Neuron	Cell Ontology + literature evidence	CL:0000101	semantic-only
L2_NG2_glia	NG2 glia	Level 2	L1_Glial_cell	literature evidence	NA	semantic-only
L2_Neural_stem_cell	Neural stem cell	Level 2	L1_Progenitor	Cell Ontology + literature evidence	CL:0000047	semantic-only
L2_Meningeal_cell	Meningeal cell	Level 2	L1_Non-neural_cell_of_nervous_system	literature evidence	NA	semantic-only
L2_Vascular_endothelial_progenitor	Vascular endothelial progenitor	Level 2	L1_Non-neural_cell_of_nervous_system	literature evidence	NA	semantic-only
L3_Amygdala_excitatory_neuron	Amygdala excitatory neuron	Level 3	L2_Excitatory_neuron	Cell Ontology + scRNA-seq atlas + literature evidence	CL:4023039	direct
L3_Astrocyte	Astrocyte	Level 3	L2_Astrocyte	Cell Ontology + scRNA-seq atlas + literature evidence	CL:0000127	direct
L3_Bergmann_glial_cell	Bergmann glial cell	Level 3	L2_Astrocyte	Cell Ontology + scRNA-seq atlas + literature evidence	CL:0000644	direct
L3_Central_nervous_system_macroph	Central nervous system macrophage	Level 3	L2_Macrophage	Cell Ontology + literature evidence	CL:0000878	semantic-only

node_id	canonical_name	level	parent_node	source	Cell_Ontology_ID	molecular_anchor_status
age						
L3_Cerebellar_inhibitory_neuron	Cerebellar inhibitory neuron	Level 3	L2_Inhibitory_neuron	scRNA-seq atlas + literature evidence	NA	direct
L3_CGE-derived_interneuron	CGE-derived interneuron	Level 3	L2_Interneuron	scRNA-seq atlas + literature evidence	NA	direct
L3_Chandelier_cell	Chandelier cell	Level 3	L2_Interneuron	Cell Ontology + scRNA-seq atlas + literature evidence	CL:4023083	direct
L3_Choroid_plexus_epithelial_cell	Choroid plexus epithelial cell	Level 3	L2_Epithelial_cell	Cell Ontology + scRNA-seq atlas + literature evidence	CL:0000706	direct
L3_Committed_oligodendrocyte_precursor	Committed oligodendrocyte precursor	Level 3	L2_Oligodendrocyte_precursor	Cell Ontology + scRNA-seq atlas + literature evidence	CL:4023059	direct
L3_Cortical_deep-layer_corticothalamic_neuron	Cortical deep-layer corticothalamic neuron	Level 3	L2_Cortical_neuron	scRNA-seq atlas + literature evidence	NA	direct
L3_Cortical_deep-layer_intratelencephalic_neuron	Cortical deep-layer intratelencephalic neuron	Level 3	L2_Cortical_neuron	scRNA-seq atlas + literature evidence	NA	direct
L3_Cortical_deep-layer_near-projecting_neuron	Cortical deep-layer near-projecting neuron	Level 3	L2_Cortical_neuron	scRNA-seq atlas + literature evidence	NA	direct
L3_Eccentric_medium_spiny_neuron	Eccentric medium spiny neuron	Level 3	L2_Medium_spiny_neuron	Cell Ontology + scRNA-seq atlas + literature evidence	CL:4030057	direct
L3_Endothelial_cell	Endothelial cell	Level 3	L2_Endothelial_cell	Cell Ontology + scRNA-seq atlas + literature evidence	CL:0000115	direct
L3_Ependymal_cell	Ependymal cell	Level 3	L2_Ependymal_cell	Cell Ontology + scRNA-seq atlas + literature evidence	CL:0000065	direct
L3_Fibroblast	Fibroblast	Level 3	L2_Fibroblast	Cell Ontology + scRNA-seq atlas + literature evidence	CL:0000057	direct
L3_Hilar_mossy_cell	Hilar mossy cell	Level 3	L2_Granule_cell	scRNA-seq atlas + literature evidence	NA	propagated
L3_Hippocampal_CA1_pyramidal_neuron	Hippocampal CA1 pyramidal neuron	Level 3	L2_Pyramidal_neuron	scRNA-seq atlas + literature evidence	NA	propagated
L3_Hippocampal_CA2_pyramidal_neuron	Hippocampal CA2 pyramidal neuron	Level 3	L2_Pyramidal_neuron	scRNA-seq atlas + literature evidence	NA	propagated
L3_Hippocampal_CA3_pyramidal_neuron	Hippocampal CA3 pyramidal neuron	Level 3	L2_Pyramidal_neuron	scRNA-seq atlas + literature evidence	NA	propagated
L3_Hippocampal_dentate_gyrus_granule_cell	Hippocampal dentate gyrus granule cell	Level 3	L2_Granule_cell	Cell Ontology + scRNA-seq atlas + literature evidence	CL:2000089	direct
L3_Interneuron	Interneuron	Level 3	L2_Interneuron	Cell Ontology + scRNA-seq atlas + literature evidence	CL:0000099	propagated
L3_Medium_spiny_neuron	Medium spiny neuron	Level 3	L2_Medium_spiny_neuron	Cell Ontology + scRNA-seq atlas + literature evidence	CL:1001474	direct
L3_MGE-derived_interneuron	MGE-derived interneuron	Level 3	L2_Interneuron	scRNA-seq atlas + literature evidence	NA	direct
L3_Microglia	Microglia	Level 3	L2_Microglia	Cell Ontology + scRNA-seq atlas + literature evidence	CL:0000129	direct
L3_Midbrain-derived_inhibitory_neuron	Midbrain-derived inhibitory neuron	Level 3	L2_Inhibitory_neuron	Cell Ontology + scRNA-seq atlas + literature evidence	CL:4023079	direct
L3_Neural_crest_cell	Neural crest cell	Level 3	L2_Neural_crest_cell	Cell Ontology + scRNA-seq atlas + literature evidence	CL:0011012	direct
L3_Neuroblast_(Vertebrate)	Neuroblast (Vertebrate)	Level 3	L2_Neuroblast	Cell Ontology + scRNA-seq atlas + literature evidence	CL:0000031	direct
L3_Neuroplacodal_cell	Neuroplacodal cell	Level 3	L2_Neuroplacodal_cell	Cell Ontology + scRNA-seq atlas + literature evidence	CL:0000032	direct
L3_Oligodendrocyte	Oligodendrocyte	Level 3	L2_Oligodendrocyte	Cell Ontology + scRNA-seq atlas + literature evidence	CL:0000128	direct
L3_Oligodendrocyte_precursor_cell	Oligodendrocyte precursor cell	Level 3	L2_Oligodendrocyte_precursor	Cell Ontology + scRNA-seq atlas + literature evidence	CL:0002453	direct
L3_Pericyte	Pericyte	Level 3	L2_Pericyte	Cell Ontology + scRNA-seq atlas + literature evidence	CL:0000669	direct
L3_Radial_glial_cell	Radial glial cell	Level 3	L2_Radial_glial_cell	Cell Ontology + scRNA-seq atlas + literature evidence	CL:0000681	direct

node_id	canonical_name	level	parent_node	source	Cell_Ontology_ID	molecular_anchor_status
L3_Thalamic_excitatory_neuron	Thalamic excitatory neuron	Level 3	L2_Excitatory_neuron	Cell Ontology + scRNA-seq atlas + literature evidence	CL:4023068	direct
L3_Vascular_smooth_muscle_cell	Vascular smooth muscle cell	Level 3	L2_Smooth_muscle_cell	scRNA-seq atlas + literature evidence	NA	direct
L3_Substantia_nigra_dopaminergic_neuron	Substantia nigra dopaminergic neuron	Level 3	L2_Dopaminergic_neuron	Cell Ontology + literature evidence	CL:4042025	semantic-only
L3_Basal_forebrain_cholinergic_neuron	Basal forebrain cholinergic neuron	Level 3	L2_Cholinergic_neuron	literature evidence	NA	semantic-only
L3_Dorsal_raphe_serotonergic_neuron	Dorsal raphe serotonergic neuron	Level 3	L2_Serotonergic_neuron	literature evidence	NA	semantic-only
L3_Dorsal_root_ganglion_neuron	Dorsal root ganglion neuron	Level 3	L2_Sensory_neuron	Cell Ontology + literature evidence	CL:1001451	semantic-only
L3_Polydendrocyte_(NG2_glia)	Polydendrocyte (NG2 glia)	Level 3	L2_NG2_glia	literature evidence	NA	semantic-only
L3_Neural_stem_cell	Neural stem cell	Level 3	L2_Neural_stem_cell	Cell Ontology + literature evidence	CL:0000047	semantic-only
L3_Dura_cell	Dura cell	Level 3	L2_Meningeal_cell	literature evidence	NA	semantic-only
L3_arachnoid_cell	arachnoid cell	Level 3	L2_Meningeal_cell	literature evidence	NA	semantic-only
L3_pia_cell	pia cell	Level 3	L2_Meningeal_cell	literature evidence	NA	semantic-only
L3_Vascular_endothelial_progenitor_cell	Vascular endothelial progenitor cell	Level 3	L2_Vascular_endothelial_progenitor	literature evidence	NA	semantic-only

Supplementary Table 1b. Definitions and per-node notes for the 79-node neural cell hierarchy

Biological definitions and per-node curation notes for each hierarchy node listed in Supplementary Table 1a. Rows are ordered identically to Table 1a and can be cross-referenced by node_id.

node_id	canonical_name	definition	notes
node_id	canonical_name	definition	notes
L1_Neuron	Neuron	The basic cellular unit of nervous tissue. Each neuron consists of a body, an axon, and dendrites.	Broad Level-1 cell class; no direct atlas annotation or molecular marker profile.
L1_Glial_cell	Glial cell	Glial cells are heterogeneous non-neuronal cells essential for neuronal support, myelination, nervous system development, and increasingly recognized roles in sensory, immune, and autonomic functions across the nervous system.	Broad Level-1 cell class; no direct atlas annotation or molecular marker profile. No direct Cell Ontology identifier; definition supported by literature/reference.
L1_Non-neural_cell_of_nervous_system	Non-neural cell of nervous system	Non-neural, non-glial cell types in the CNS of mesenchymal, endothelial, or epithelial origin.	Broad Level-1 cell class; no direct atlas annotation or molecular marker profile. No direct Cell Ontology identifier; definition supported by literature/reference.
L1_Progenitor	Progenitor	Progenitor cells in the nervous system are self-renewing, multipotent precursors that generate neurons, glia, and related cell types, including both central (CNS) and peripheral (PNS) lineages.	Broad Level-1 cell class; no direct atlas annotation or molecular marker profile. No direct Cell Ontology identifier; definition supported by literature/reference.
L2_Excitatory_neuron	Excitatory neuron	A glutamatergic neuron that enhances postsynaptic activity; originates in the dorsal ventricular zone and populates cortical and subcortical regions.	No direct atlas annotation at L2; molecular context aggregated from transcriptomically supported L3 child node(s). No direct Cell Ontology identifier; definition supported by literature.
L2_Astrocyte	Astrocyte	A star-shaped macroglial cell in the CNS; the most abundant glial type. Regulates the extracellular environment, contributes to the blood-brain barrier via end feet, and becomes reactive in response to injury.	L2 shares name with a directly resolved L3 child; molecular context from same-name L3 node.
L2_Macrophage	Macrophage	Mononuclear phagocyte: tissue-resident phagocyte derived from monocytes; clears microbes and debris.	No direct atlas annotation; retained as ontology/literature-supported hierarchy node.
L2_Inhibitory_neuron	Inhibitory neuron	A GABAergic or glycinergic neuron that suppresses postsynaptic firing; derived from ventral ganglionic eminences and integrates into diverse brain circuits to maintain excitation-inhibition balance.	No direct atlas annotation at L2; molecular context aggregated from transcriptomically supported L3 child node(s). No direct Cell Ontology identifier; definition supported by literature.
L2_Interneuron	Interneuron	Most generally any neuron which is not motor or sensory. Interneurons may also refer to neurons whose axons remain within a particular brain region as contrasted with projection neurons which have axons projecting to other brain regions.	No direct atlas annotation at L2; molecular context aggregated from transcriptomically supported L3 child node(s).
L2_Epithelial_cell	Epithelial cell	A cell that is usually found in a two-dimensional sheet with a free surface. The cell has a cytoskeleton that allows for tight cell to cell contact and for cell polarity where apical part is	No direct atlas annotation at L2; molecular context aggregated from transcriptomically supported L3 child node(s).

node_id	canonical_name	definition	notes
		directed toward the lumen and the basal part to the basal lamina.	
L2_Oligodendrocyte_precursor	Oligodendrocyte precursor	A proliferative and migratory glial progenitor cell that derives from a neural stem cell and resides within the central nervous system.	No direct atlas annotation at L2; molecular context aggregated from transcriptomically supported L3 child node(s).
L2_Cortical_neuron	Cortical neuron	Cerebral cortex cell that processes sensory input and generates motor output; divides into glutamatergic projection neurons and local GABAergic interneurons, forming the circuits underlying cognition, perception and consciousness.	No direct atlas annotation at L2; molecular context aggregated from transcriptomically supported L3 child node(s).
L2_Medium_spiny_neuron	Medium spiny neuron	An inhibitory, GABAergic projection neuron in the striatum that integrates glutamatergic signals arising from the cerebral cortex and thalamus.	L2 shares name with a directly resolved L3 child; molecular context from same-name L3 node.
L2_Endothelial_cell	Endothelial cell	An endothelial cell comprises the outermost layer or lining of anatomical structures and can be squamous or cuboidal. In mammals, endothelial cell has vimentin filaments and is derived from the mesoderm.	L2 shares name with a directly resolved L3 child; molecular context from same-name L3 node.
L2_Ependymal_cell	Ependymal cell	A neuroepithelial glial cell, derived from a radial glial cell originating from the neuroectoderm, lines the ventricles of the brain and the central canal of the spinal cord. This cell is characterized by the presence of cilia on its apical surface, which can be motile or non-motile.	L2 shares name with a directly resolved L3 child; molecular context from same-name L3 node.
L2_Fibroblast	Fibroblast	A connective tissue cell which secretes an extracellular matrix rich in collagen and other macromolecules. Flattened and irregular in outline with branching processes; appear fusiform or spindle-shaped.	L2 shares name with a directly resolved L3 child; molecular context from same-name L3 node.
L2_Granule_cell	Granule cell	A neuron of the vertebrate central nervous system that is small in size. This general class includes small neurons in the granular layer of the cerebellar cortex, cerebral cortex neurons that are not pyramidal cells and small neurons without axons found in the olfactory bulb.	No direct atlas annotation at L2; molecular context aggregated from transcriptomically supported L3 child node(s).
L2_Pyramidal_neuron	Pyramidal neuron	Pyramidal neurons have a pyramid-shaped soma with a single axon, a large apical dendrite and multiple basal dendrites. The apex and an apical dendrite typically point toward the pial surface and other dendrites and an axon emerging from the base.	Atlas annotation category mapped directly to this L2 node (combined entry); markers attached at L2 level.
L2_Microglia	Microglia	Yolk-sac-derived resident macrophages of the central nervous system, comprising 10% of brain cells. In the healthy state they display a ramified morphology; upon injury or infection they transform into amoeboid phagocytes.	L2 shares name with a directly resolved L3 child; molecular context from same-name L3 node.
L2_Neural_crest_cell	Neural crest cell	A cell of the neural crest. Neural crest cells are multipotent.	L2 shares name with a directly resolved L3 child; molecular context from same-name L3 node.
L2_Neuroblast	Neuroblast	A cell that will develop into a	No direct atlas annotation at L2;

node_id	canonical_name	definition	notes
		neuron often after a migration phase.	molecular context aggregated from transcriptomically supported L3 child node(s).
L2_Neuroplacodal_cell	Neuroplacodal cell	A cell of a platelike structure, especially a thickened plate of ectoderm in the early embryo, from which a sense organ develops.	L2 shares name with a directly resolved L3 child; molecular context from same-name L3 node.
L2_Oligodendrocyte	Oligodendrocyte	A class of large neuroglial (macroglial) cells in the central nervous system. Form the insulating myelin sheath of axons in the central nervous system.	L2 shares name with a directly resolved L3 child; molecular context from same-name L3 node.
L2_Pericyte	Pericyte	An elongated, contractile cell found wrapped about precapillary arterioles outside the basement membrane. Pericytes are present in capillaries where proper adventitia and muscle layer are missing (thus distinguishing this cell type from adventitial cells).	L2 shares name with a directly resolved L3 child; molecular context from same-name L3 node.
L2_Radial_glial_cell	Radial glial cell	A cell present in the developing CNS. Functions as both a precursor cell and as a scaffold to support neuronal migration.	L2 shares name with a directly resolved L3 child; molecular context from same-name L3 node.
L2_Smooth_muscle_cell	Smooth muscle cell	A non-striated, elongated, spindle-shaped cell found lining the digestive tract, uterus, and blood vessels. They develop from specialized myoblasts (smooth muscle myoblast).	No direct atlas annotation at L2; molecular context aggregated from transcriptomically supported L3 child node(s).
L2_Dopaminergic_neuron	Dopaminergic neuron	A neuron that releases dopamine as a neurotransmitter.	No direct atlas annotation; retained as ontology/literature-supported hierarchy node.
L2_Cholinergic_neuron	Cholinergic neuron	A neuron that uses acetylcholine as a vesicular neurotransmitter.	No direct atlas annotation; retained as ontology/literature-supported hierarchy node.
L2_Serotonergic_neuron	Serotonergic neuron	A neuron that releases serotonin as a neurotransmitter.	No direct atlas annotation; retained as ontology/literature-supported hierarchy node.
L2_Sensory_neuron	Sensory neuron	Any neuron having a sensory function; an afferent neuron conveying sensory impulses.	No direct atlas annotation; retained as ontology/literature-supported hierarchy node.
L2_NG2_glia	NG2 glia	NG2-expressing glial cells in the CNS; function as oligodendrocyte progenitors and active modulators of neural circuits through neuron-glia interactions, neuromodulation, and neuroprotection.	No direct atlas annotation; retained as ontology/literature-supported hierarchy node. No direct Cell Ontology identifier; definition supported by literature.
L2_Neural_stem_cell	Neural stem cell	An undifferentiated neural cell that originates from the neuroectoderm and has the capacity both to perpetually self-renew without differentiating and to generate multiple central nervous system neuronal and glial cell types.	No direct atlas annotation; retained as ontology/literature-supported hierarchy node.
L2_Meningeal_cell	Meningeal cell	Structural cell of the three meningeal layers (dura, arachnoid, pia) that secretes collagen and basement-membrane components, forms the epithelial-like arachnoid barrier, and acts as an essential interface between CNS and periphery during development, homeostasis and disease.	No direct atlas annotation; retained as ontology/literature-supported hierarchy node. No direct Cell Ontology identifier; definition supported by literature.
L2_Vascular_endothelial_progenitor	Vascular endothelial progenitor	Rare, highly proliferative cell committed to endothelial lineage; capable of colony formation and vascular integration; mediates vessel repair chiefly via paracrine	No direct atlas annotation; retained as ontology/literature-supported hierarchy node. No direct Cell Ontology identifier; definition supported by literature.

node_id	canonical_name	definition	notes
		signals rather than long-term engraftment.	
L3_Amygdala_excitatory_neuron	Amygdala excitatory neuron	Any neuron that has its soma located in some amygdala and is capable of some glutamate secretion, neurotransmission.	Directly resolved in reference scRNA-seq atlas with marker genes identified under differential-expression criteria.
L3_Astrocyte	Astrocyte	Star-shaped glial cell with end feet contributing to the blood-brain barrier; regulates extracellular homeostasis and becomes reactive after injury.	Directly resolved in reference scRNA-seq atlas with marker genes identified under differential-expression criteria.
L3_Bergmann_glial_cell	Bergmann glial cell	Cerebellar radial astrocyte that spans Purkinje to pia, buffers glutamate and sculpts synapses.	Directly resolved in reference scRNA-seq atlas with marker genes identified under differential-expression criteria.
L3_Central_nervous_system_macrophage	Central nervous system macrophage	A tissue-resident macrophage found in the central nervous system.	No atlas annotation or molecular marker profile; retained as literature-supported semantic anchor.
L3_Cerebellar_inhibitory_neuron	Cerebellar inhibitory neuron	Cerebellar inhibitory interneurons are GABAergic and/or glycinergic neurons derived from Ptf1a-expressing progenitors in the embryonic cerebellar ventricular neuroepithelium.	Directly resolved in reference scRNA-seq atlas with marker genes identified under differential-expression criteria. No direct Cell Ontology identifier; definition supported by literature.
L3_CGE-derived_interneuron	CGE-derived interneuron	GABAergic neuron born in the caudal ganglionic eminence;accounts for 50% of human cortical inhibitory neurons; mediates disinhibition and cognitive processing.	Directly resolved in reference scRNA-seq atlas with marker genes identified under differential-expression criteria. No direct Cell Ontology identifier; definition supported by literature.
L3_Chandelier_cell	Chandelier cell	A GABAergic interneuron that selectively innervates the axon initial segment of pyramidal cells. Their local axonal clusters are formed by high-frequency branching at shallow angles, often ramifying around, above or below their somata with a high bouton density.	Directly resolved in reference scRNA-seq atlas with marker genes identified under differential-expression criteria.
L3_Choroid_plexus_epithelial_cell	Choroid plexus epithelial cell	A specialized ependymal cell that is part of the choroid plexus epithelium, responsible for producing cerebrospinal fluid (CSF) by selectively filtering and modifying blood plasma components before secreting it into the brain and spinal cord.	Directly resolved in reference scRNA-seq atlas with marker genes identified under differential-expression criteria.
L3_Committed_oligodendrocyte_precursor	Committed oligodendrocyte precursor	An oligodendrocyte precursor cell that is committed to differentiate.	Directly resolved in reference scRNA-seq atlas with marker genes identified under differential-expression criteria.
L3_Cortical_deep-layer_corticothalamic_neuron	Cortical deep-layer corticothalamic neuron	L5 thick-tufted pyramidal neuron projecting to thalamus, superior colliculus and brainstem; intrinsically bursting, direction-selective, high-temporal-frequency preference.	Directly resolved in reference scRNA-seq atlas with marker genes identified under differential-expression criteria. No direct Cell Ontology identifier; definition supported by literature.
L3_Cortical_deep-layer_intratelencephalic_neuron	Cortical deep-layer intratelencephalic neuron	L5 thin-tufted pyramidal neuron with bilateral cortical and striatal axons; regular-spiking, intermediate spatial-frequency tuning, mediates inter-hemispheric feedforward.	Directly resolved in reference scRNA-seq atlas with marker genes identified under differential-expression criteria. No direct Cell Ontology identifier; definition supported by literature.
L3_Cortical_deep-layer_near-projecting_neuron	Cortical deep-layer near-projecting neuron	L5 oval-soma pyramidal neuron restricted to adjacent ipsilateral cortex, no striatal/subcortical collaterals; high input resistance, high spatial-frequency preference, local output channel.	Directly resolved in reference scRNA-seq atlas with marker genes identified under differential-expression criteria. No direct Cell Ontology identifier; definition supported by literature.
L3_Eccentric_medium_spiny_ne	Eccentric medium spiny neuron	A medium spiny neuron that	Directly resolved in reference

node_id	canonical_name	definition	notes
uron		exhibits transcriptional divergence from direct and indirect spiny projection neurons, for example, enrichment in Casz1, Otof, Cacng5 and Pcdh8 noted in mice.	scRNA-seq atlas with marker genes identified under differential-expression criteria.
L3_Endothelial_cell	Endothelial cell	An endothelial cell comprises the outermost layer or lining of anatomical structures and can be squamous or cuboidal. In mammals, endothelial cell has vimentin filaments and is derived from the mesoderm.	Directly resolved in reference scRNA-seq atlas with marker genes identified under differential-expression criteria.
L3_Ependymal_cell	Ependymal cell	A neuroepithelial glial cell, derived from a radial glial cell originating from the neuroectoderm, lines the ventricles of the brain and the central canal of the spinal cord. This cell is characterized by the presence of cilia on its apical surface, which can be motile or non-motile.	Directly resolved in reference scRNA-seq atlas with marker genes identified under differential-expression criteria.
L3_Fibroblast	Fibroblast	A connective tissue cell which secretes an extracellular matrix rich in collagen and other macromolecules. Flattened and irregular in outline with branching processes; appear fusiform or spindle-shaped.	Directly resolved in reference scRNA-seq atlas with marker genes identified under differential-expression criteria.
L3_Hilar_mossy_cell	Hilar mossy cell	A glutamatergic principal neuron located in the polymorphic hilus of the dentate gyrus that receives excitatory input from granule cells and provides extensive feedback projections to both granule cells and local GABAergic interneurons.	Not independently resolved in atlas; molecular context propagated from transcriptomically supported parent node. No direct Cell Ontology identifier; definition supported by literature.
L3_Hippocampal_CA1_pyramidal_neuron	Hippocampal CA1 pyramidal neuron	Hippocampal CA1 pyramidal neurons are glutamatergic principal neurons located in the CA1 subfield of the hippocampus that play a central role in spatial navigation and mnemonic processing.	Atlas resolved hippocampal pyramidal neurons as a combined 'Hippocampal CA1, CA2, and CA3 pyramidal neuron' annotation category (markers attached to L2 Pyramidal neuron); CA1 subregion not independently resolved. Not independently resolved in atlas; molecular context propagated from transcriptomically supported parent node. No direct Cell Ontology identifier; definition supported by literature.
L3_Hippocampal_CA2_pyramidal_neuron	Hippocampal CA2 pyramidal neuron	Hippocampal CA2 pyramidal neurons are a distinct subpopulation of glutamatergic principal neurons located in the CA2 subfield of the hippocampus.	Atlas resolved hippocampal pyramidal neurons as a combined 'Hippocampal CA1, CA2, and CA3 pyramidal neuron' annotation category (markers attached to L2 Pyramidal neuron); CA2 subregion not independently resolved. Not independently resolved in atlas; molecular context propagated from transcriptomically supported parent node. No direct Cell Ontology identifier; definition supported by literature.
L3_Hippocampal_CA3_pyramidal_neuron	Hippocampal CA3 pyramidal neuron	An excitatory projection neuron located in the stratum pyramidale of the hippocampal CA3 region, characterized by dense recurrent collateral axons that form an autoassociative network.	Atlas resolved hippocampal pyramidal neurons as a combined 'Hippocampal CA1, CA2, and CA3 pyramidal neuron' annotation category (markers attached to L2 Pyramidal neuron); CA3

node_id	canonical_name	definition	notes
			subregion not independently resolved. Not independently resolved in atlas; molecular context propagated from transcriptomically supported parent node. No direct Cell Ontology identifier; definition supported by literature.
L3_Hippocampal_dentate_gyrus_granule_cell	Hippocampal dentate gyrus granule cell	A granule cell that has soma location in the dentate gyrus cell layer of the hippocampal formation and has an elliptical cell body and characteristic cone-shaped tree of spiny apical dendrites.	Directly resolved in reference scRNA-seq atlas with marker genes identified under differential-expression criteria.
L3_Interneuron	Interneuron	Most generally any neuron which is not motor or sensory. Interneurons may also refer to neurons whose axons remain within a particular brain region as contrasted with projection neurons which have axons projecting to other brain regions.	Generic L3 interneuron node; molecular context propagated from L2 Interneuron (which aggregates specific interneuron subtypes with direct atlas support). Not independently resolved in atlas; molecular context propagated from transcriptomically supported parent node.
L3_Medium_spiny_neuron	Medium spiny neuron	An inhibitory, GABAergic projection neuron in the striatum that integrates glutamatergic signals arising from the cerebral cortex and thalamus.	Directly resolved in reference scRNA-seq atlas with marker genes identified under differential-expression criteria.
L3_MGE-derived_interneuron	MGE-derived interneuron	MGE-derived interneurons are a transient embryonic population born in the medial ganglionic eminence (MGE) that express the transcription factors Lhx6 and Nkx2.1, migrate to cortex and striatum between E12.5--P10.	Directly resolved in reference scRNA-seq atlas with marker genes identified under differential-expression criteria. No direct Cell Ontology identifier; definition supported by literature.
L3_Microglia	Microglia	Microglia are the primary resident immune cells of the central nervous system (CNS), constituting approximately 10% of all CNS cells. Originating from yolk sac progenitors, they differ from other glial cells (such as astrocytes and oligodendrocytes), which arise from neuroectodermal lineages.	Directly resolved in reference scRNA-seq atlas with marker genes identified under differential-expression criteria.
L3_Midbrain-derived_inhibitory_neuron	Midbrain-derived inhibitory neuron	A GABAergic inhibitory neuron that is derived from the midbrain.	Directly resolved in reference scRNA-seq atlas with marker genes identified under differential-expression criteria.
L3_Neural_crest_cell	Neural crest cell	A cell of the neural crest. Neural crest cells are multipotent.	Directly resolved in reference scRNA-seq atlas with marker genes identified under differential-expression criteria.
L3_Neuroblast_(Vertebrate)	Neuroblast (Vertebrate)	A cell that will develop into a neuron often after a migration phase.	Directly resolved in reference scRNA-seq atlas with marker genes identified under differential-expression criteria.
L3_Neuroplacodal_cell	Neuroplacodal cell	A cell of a platelike structure, especially a thickened plate of ectoderm in the early embryo, from which a sense organ develops.	Directly resolved in reference scRNA-seq atlas with marker genes identified under differential-expression criteria.
L3_Oligodendrocyte	Oligodendrocyte	A class of large neuroglial (macroglial) cells in the central nervous system. Form the insulating myelin sheath of axons in the central nervous system.	Directly resolved in reference scRNA-seq atlas with marker genes identified under differential-expression criteria.
L3_Oligodendrocyte_precursor_cell	Oligodendrocyte precursor cell	A proliferative and migratory glial progenitor cell that derives from a neural stem cell and resides within the central nervous system.	Directly resolved in reference scRNA-seq atlas with marker genes identified under differential-expression criteria.

node_id	canonical_name	definition	notes
L3_Pericyte	Pericyte	An elongated, contractile cell found wrapped about precapillary arterioles outside the basement membrane. Pericytes are present in capillaries where proper adventitia and muscle layer are missing (thus distinguishing this cell type from adventitial cells).	Directly resolved in reference scRNA-seq atlas with marker genes identified under differential-expression criteria.
L3_Radial_glial_cell	Radial glial cell	A cell present in the developing CNS. Functions as both a precursor cell and as a scaffold to support neuronal migration.	Directly resolved in reference scRNA-seq atlas with marker genes identified under differential-expression criteria.
L3_Thalamic_excitatory_neuron	Thalamic excitatory neuron	An excitatory neuron that has its soma located in the thalamic complex. This neuron type can be involved in a series of circuits related to sensory integration, motor integration, pain processing, social behavior and reward response.	Directly resolved in reference scRNA-seq atlas with marker genes identified under differential-expression criteria.
L3_Vascular_smooth_muscle_cell	Vascular smooth muscle cell	Vascular smooth muscle cells (VSMCs) are the predominant cellular component of the medial layer of blood vessel walls, responsible for maintaining vascular tone, elasticity, and structural integrity.	Directly resolved in reference scRNA-seq atlas with marker genes identified under differential-expression criteria. No direct Cell Ontology identifier; definition supported by literature.
L3_Substantia_nigra_dopaminergic_neuron	Substantia nigra dopaminergic neuron	A midbrain dopaminergic neuron that has its soma located in a substantia nigra. This dopaminergic neuron type is highly metabolically active and it is involved in the regulation of movement, cognition, motivation and reward.	No atlas annotation or molecular marker profile; retained as literature-supported semantic anchor.
L3_Basal_forebrain_cholinergic_neuron	Basal forebrain cholinergic neuron	Basal forebrain cholinergic neurons (BFCNs) are magnocellular, acetylcholine-producing neurons forming a discontinuous band from septum to substantia innominata and nucleus basalis, projecting densely to cortex.	No atlas annotation or molecular marker profile; retained as literature-supported semantic anchor. No direct Cell Ontology identifier; definition supported by literature.
L3_Dorsal_raphe_serotonergic_neuron	Dorsal raphe serotonergic neuron	Dorsal raphe serotonergic neurons (DRN 5-HT neurons) are serotonin-producing cells located in the dorsal raphe nucleus that project widely to forebrain regions.	No atlas annotation or molecular marker profile; retained as literature-supported semantic anchor. No direct Cell Ontology identifier; definition supported by literature.
L3_Dorsal_root_ganglion_neuron	Dorsal root ganglion neuron	A sensory neuron of the dorsal root ganglia that senses body position and sends information about how much the muscle is stretched to the spinal cord.	No atlas annotation or molecular marker profile; retained as literature-supported semantic anchor.
L3_Polydendrocyte_(NG2_glia)	Polydendrocyte (NG2 glia)	Polydendrocytes (NG2 glia) are a distinct resident glial population that uniformly occupy gray and white matter of the developing and adult CNS, express NG2 proteoglycan and PDGF receptor.	No atlas annotation or molecular marker profile; retained as literature-supported semantic anchor. No direct Cell Ontology identifier; definition supported by literature.
L3_Neural_stem_cell	Neural stem cell	An undifferentiated neural cell that originates from the neuroectoderm and has the capacity both to perpetually self-renew without differentiating and to generate multiple central nervous system neuronal and glial cell types.	No atlas annotation or molecular marker profile; retained as literature-supported semantic anchor.
L3_Dura_cell	Dura cell	Dura cells constitute the outermost meningeal fibroblast population lining the inner calvarial surface and are essential for intracranial mechanical homeostasis and	No atlas annotation or molecular marker profile; retained as literature-supported semantic anchor. No direct Cell Ontology identifier; definition supported by literature.

node_id	canonical_name	definition	notes
		immune surveillance.	
L3_arachnoid_cell	arachnoid cell	The intermediate layer of epithelial-like cells located between the dura mater and pia mater highly expresses SLC22A6/SLC22A2 and CDH1, and forms the arachnoid barrier through dense tight junctions (CLDNs, OCLN, JAMs, etc.), physically separating the cerebrospinal fluid from the venous blood.	No atlas annotation or molecular marker profile; retained as literature-supported semantic anchor. No direct Cell Ontology identifier; definition supported by literature.
L3_pia_cell	pia cell	The innermost layer of flattened fibroblast-like cells, closely apposed to the surface of the brain-spinal cord parenchyma, originates from the embryonic primary meninx and specifically expresses LAMC3.	No atlas annotation or molecular marker profile; retained as literature-supported semantic anchor. No direct Cell Ontology identifier; definition supported by literature.
L3_Vascular_endothelial_progenitor_cell	Vascular endothelial progenitor cell	Vascular endothelial progenitor cells are a class of precursor cells that possess the capacity for self-renewal and the potential to differentiate into functional blood vessels with an intimal layer.	No atlas annotation or molecular marker profile; retained as literature-supported semantic anchor. No direct Cell Ontology identifier; definition supported by literature.

Supplementary Table 2. CellMarker-based validation of transcriptome-derived molecular fingerprints

**Cell type**	**Topgenes**	**Proteingenes**	**CMgenes**	**Specificmatches**	**Specifichit (%)**	**Brain-widematches**	**Brain-widehit (%)**
Cell type	Topgenes	Proteingenes	CMgenes	Specificmatches	Specifichit (%)	Brain-widematches	Brain-widehit (%)
Amygdala excitatory neuron	50	29	175	2	6.9	6	20.7
Astrocyte	50	35	214	12	34.3	16	45.7
Bergmann glia cell	50	46	211	8	17.4	16	34.8
CGE-derived interneuron	50	33	14	0	0.0	12	36.4
Cerebellar inhibitory	45	30	167	2	6.7	7	23.3
Chandelier cell	48	38	11	0	0.0	12	31.6
Choroid plexus epithelial cell	19	12	1	0	0.0	3	25.0
Committed oligodendrocyte precursor	50	38	17	4	10.5	19	50.0
Cortical deep-layer corticothalamic neuron	50	29	175	1	3.4	8	27.6
Cortical deep-layer intratelencephalic neuron	50	18	175	0	0.0	8	44.4
Cortical deep-layer near-projecting neuron	45	30	175	0	0.0	2	6.7
Eccentric medium spiny neuron	50	34	167	3	8.8	4	11.8
Endothelial cell	50	48	24	8	16.7	14	29.2
Ependymal cell	19	15	3	1	6.7	1	6.7
Fibroblast	50	47	10	4	8.5	13	27.7
Hippocampal CA1,CA2,and CA3 pyramidal neuron	50	28	176	1	3.6	5	17.9
Hippocampal CA4 pyramidal neuron	40	25	176	1	4.0	4	16.0
Hippocampal dentate gyrus granule cell	32	15	175	0	0.0	1	6.7
MGE-derived interneuron	50	37	16	2	5.4	19	51.4
Medium spiny neuron	50	28	167	1	3.6	10	35.7
Microglia	50	47	349	19	40.4	26	55.3
Midbrain-derived inhibitory neuron	27	18	171	0	0.0	2	11.1
Neural crest cell	30	26	7	1	3.8	11	42.3
Neuroblast (Vertebrate)	50	44	24	0	0.0	13	29.5
Neuroplacodal cell	50	45	4	0	0.0	9	20.0
Oligodendrocyte	50	28	177	6	21.4	9	32.1
Oligodendrocyte precursor cell	50	43	17	8	18.6	20	46.5
Pericyte	50	42	19	2	4.8	6	14.3
Radial glia cell	50	39	33	1	2.6	8	20.5
Thalamic excitatory neuron	50	35	175	1	2.9	4	11.4
Vascular smooth muscle cell	43	40	7	3	7.5	9	22.5

The table summarizes overlap between study-derived marker candidates and CellMarker 2.0 annotations. “Specific matches” indicates overlap with the corresponding CellMarker cell-type entry; ``brain-wide matches" indicates overlap with any brain-related CellMarker entry.

Supplementary Table 3. Canonical marker recovery in Top 50 candidate lists with stratified analysis

**Cell type**	**No.tested**	**No.recovered**	**Recovery(%)**	**Category**	**Recovered markers**	**MissingCellMarker markers**	**Interpretation**
Cell type	No.tested	No.recovered	Recovery(%)	Category	Recovered markers	MissingCellMarker markers	Interpretation
Endothelial cell	6	6	100.0	Perfect	CLDN5, FLT1, VWF, PECAM1, CDH5, ERG	--	Canonical markers fully captured; atlas annotation and DE power sufficient.
Oligodendrocyte precursor cell	5	5	100.0	Perfect	PDGFRA, CSPG4, OLIG1, OLIG2, VCAN	--	Canonical markers fully captured; atlas annotation and DE power sufficient.
Pericyte	4	4	100.0	Perfect	PDGFRB, RGS5, NOTCH3, ABCC9	--	Canonical markers fully captured; atlas annotation and DE power sufficient.
Vascular smooth muscle cell	5	5	100.0	Perfect	MYH11, ACTA2, CNN1, TAGLN, MYL9	--	Canonical markers fully captured; atlas annotation and DE power sufficient.
Oligodendrocyte	6	5	83.3	High	MOG, MBP, PLP1, MAG, MOBP	OLIG2	OLIG2 is a lineage TF shared with OPC; not specific to mature oligodendrocytes in one-versus-rest DE.
Astrocyte	6	4	66.7	High	GFAP, AQP4, SLC1A2, FGFR3	S100B, ALDH1L1	S100B and ALDH1L1 are pan-astrocyte markers with broad expression; may not pass one-versus-rest specificity threshold.
MGE-derived interneuron	3	2	66.7	High	SST, LHX6	PVALB	PVALB is a pan-interneuron marker shared with other interneuron subtypes.
Neuroblast (Vertebrate)	6	4	66.7	High	NEUROD2, NEUROG2, TUBB3, STMN2	DCX, NEUROD1	DCX and NEUROD1 are expressed across multiple neuronal precursors; may not be specific in one-versus-rest design.
Thalamic excitatory neuron	3	2	66.7	High	SLC17A6, SHOX2	TCF7L2	TCF7L2 is a thalamic identity TF; may not pass DE threshold in whole-brain atlas.
Microglia	8	5	62.5	High	CX3CR1, P2RY12, C1QA, C1QB, C1QC	TMEM119, AIF1, CSF1R	TMEM119, AIF1 and CSF1R have lower specificity in one-versus-rest DE; CSF1R is broadly expressed across immune cells.
CGE-derived	4	2	50.0	High	VIP, CXCL14	SP8, HTR3A	SP8 and HTR3A

Cell type	No.tested	No.recovered	Recovery(%)	Category	Recovered markers	MissingCellMarker markers	Interpretation
interneuron							are CGE interneuron TFs; may not pass DE threshold in whole-brain atlas.
Chandelier cell	4	2	50.0	High	LHX6, LAMP5	PVALB, NKX2-1	PVALB is shared with other interneurons; NKX2-1 is a developmental TF with transient expression.
Radial glia cell	6	3	50.0	High	NES, HES1, FABP7	SOX2, PAX6, VIM	SOX2, PAX6 and VIM are neural stem cell markers shared with other progenitor types.
Ependymal cell	5	2	40.0	Partial	CFAP299, CAPS	FOXJ1, DNAH11, SPAG6	Only 19 DE genes passed thresholds (vs 50 in most types), indicating low cell count; ciliated cell markers overlap with choroid plexus.
Medium spiny neuron	5	2	40.0	Partial	DRD2, PENK	DRD1, PPP1R1B, TAC1	Striatal neuron underrepresented in whole-brain atlas; D1 (DRD1) and D2 subtypes not distinguished.
Neural crest cell	5	2	40.0	Partial	SNAI2, TWIST1	SOX10, FOXD3, PAX3	Developmental transient population; TFs expressed at low levels; sparse in adult brain atlas.
Choroid plexus epithelial cell	4	1	25.0	Partial	CLIC6	TTR, FOLR1, KL	Only 19 DE genes passed thresholds, indicating low cell count; rare cell type with sparse atlas annotation.
Bergmann glia cell	4	0	0.0	None	--	GFAP, AQP4, HOPX, BCAN	Cerebellar astrocyte subtype; canonical markers (GFAP, AQP4) shared with general astrocytes; atlas may not resolve Bergmann glia separately.
Fibroblast	5	0	0.0	None	--	COL1A1, COL1A2, PDGFRA, LUM, DCN	Non-neural cell type poorly annotated in brain atlas; PDGFRA assigned to OPC in one-versus-rest DE.
Hippocampal CA4 pyramidal neuron	2	0	0.0	None	--	SLC17A7, SLC17A6	Not included in final hierarchy; atlas combined CA1--CA3. Excluded from the 20-type recovery analysis.
Hippocampal dentate gyrus granule cell	3	0	0.0	None	--	PROX1, CALB1, TRPC6	Only 32 DE genes; atlas may group DG granule cells under broader granule cell or hippocampal

Cell type	No.tested	No.recovered	Recovery(%)	Category	Recovered markers	MissingCellMarker markers	Interpretation
							neuron category.

Canonical marker recovery was used as an interpretable validation of the cell-type-specific marker-ranking procedure. Recovery categories were defined as Perfect (100%), High (50% and <100%), Partial (>0% and <50%) and None (0%). Cell types with low or no recovery typically had one or more of the following: low cell count in the reference atlas (yielding fewer than 50 DE genes), canonical markers shared with broader cell categories (reducing one-versus-rest specificity), or non-neural / developmental cell types with sparse atlas annotation.

Supplementary Table 4a. Inter-annotator agreement and extraction accuracy for literature-derived triples

Evaluation category	Metric	Value
Inter-annotator agreement	Overall --- Raw agreement	99.7% (330/331)
	Overall --- Cohen's	0.955
	Dopaminergic neuron --- Raw /	99.0%; 0.928
	Microglia --- Raw /	100.0%; 1.000
	Oligodendrocyte precursor --- Raw /	100.0%; 1.000
	Dorsal raphe serotonergic --- Raw /	100.0%; 1.000
	Chandelier cell --- Raw /	100.0%; 1.000
	Pia cell --- Raw /	100.0%; 1.000
Extraction accuracy	Dopaminergic neuron	92.0% (92/100)
	Microglia	98.0% (98/100)
	Oligodendrocyte precursor cell	100.0% (50/50)
	Dorsal raphe serotonergic neuron	100.0% (50/50)
	Chandelier cell	91.3% (21/23)
	Pia cell	100.0% (8/8)
	Overall	96.4% (319/331)
Dimension-specific accuracy	Entity recognition	99.4%
	Cell type mapping	98.8%
	Relation logic	98.5%
Error source distribution	Type A: entity recognition	2 (18.2%)
	Type B: cell type mapping	4 (36.4%)
	Type C: relation logic	5 (45.5%)

The 331-record expert-validation set, spanning six cell types (dopaminergic neuron, microglia, oligodendrocyte precursor cell, dorsal raphe serotonergic neuron, chandelier cell and pia cell), and the prompt templates used for screening and extraction are included in the release package as supplementary/validation_samples.csv and supplementary/llm_prompts.txt. The validation file records the extracted factor, perturbation class, action, effect, effect direction, cell type, confidence score, two independent expert labels, two error labels and the resolved gold label. Type A errors indicate incorrect factor/entity recognition, Type B errors indicate incorrect cell-type mapping, and Type C errors indicate incorrect relation logic. Per-cell raw agreement and Cohen's are reported above; full per-cell dimension-specific and error-source breakdowns are provided in Supplementary Tables 4a and 4b.

Supplementary Table 4b. Qualitative analysis of relation-logic errors (Type C)

PMID	Cell type	Factor	Error pattern	Orig. dir.	Correction
41360225	Dopaminergic neuron	MPTP	Direction reversal: neurodegeneration encoded as upregulation	+1	Reversed to -1
41520969	Dopaminergic neuron	JNK activation	Direction reversal: abolished neuroprotection encoded as upregulation	+1	Reversed to -1
41542562	Dopaminergic neuron	LAG3 knockout	Causal-polarity confusion: KO preserving TH encoded as negative	-1	Reclassified as protective (+1)
8035215	Chandelier cell	Depolarizing current injection	Non-regulatory: acute electrophysiological response	+1	Removed from REGULATES
37922650	Microglia	LPS	Non-regulatory: downstream phosphorylation cascade	+1	Reclassified as signaling event

Five relation-logic errors (Type C) were identified across the 331-record validation set. Three recurrent patterns were observed: direction reversal (n=2), causal-polarity confusion in protective contexts (n=1), and non-regulatory events misclassified as REGULATES edges (n=2). Errors spanned chemical, genetic, microbiome and physical perturbations without concentration on a single factor class, with weak enrichment in dopaminergic-neuron contexts (3/5). All cases were corrected during manual curation as indicated in the rightmost column. The overall Type C error rate of 5/331 (1.5%) provides a lower-bound estimate of residual relation-logic error in the REGULATES set after curation.

Supplementary Methods 1. LLM screening, extraction and source-data release

PubMed abstracts were processed in two LLM-assisted stages. In the screening stage, the model was instructed to return JSON only and to mark an abstract as relevant only when it explicitly mentioned a target neural cell-type term, a molecular, environmental or other perturbing factor, and a downstream molecular or cellular change in the same cell context. In the extraction stage, the model was instructed to extract one perturbation--effect relationship per record using a fixed JSON schema containing PMID, factor name, factor type, perturbation action, cell type, effect, effect direction, evidence sentence and confidence score. Both stages used temperature 0. The extraction prompt explicitly prohibited inference beyond the abstract text and required controlled vocabulary terms for perturbation-factor categories. The released regulatory-evidence table contains 25,812 extracted records from 7,461 unique PMIDs. The table fields are pmid, factor_name, factor_type_level1, factor_type_level2, perturbation_action, cell_type_mentioned, cell_type_mapping, cell_type_granularity, effect, effect_direction, evidence_sentence, confidence_score and extraction_level. The record counts by extraction level are 544 at Level 1, 6,547 at Level 2 and 18,721 at Level 3. The dominant factor categories are genetic and molecular perturbations, pharmacological and small-molecule perturbations, biological signaling molecules, environmental factors, metabolic and nutrient stress, cellular interactions, microbiome-related perturbations, radiation and energy stress, mechanical factors and metal ions. Confidence scores were used only for prioritization and quality control; expert validation was performed separately as summarized in Supplementary Table 4a.

Supplementary Table 5. Complete graph and KGE export statistics

**Scope**	**Item**	**Count**
Scope	Item	Count
Complete Neo4j graph	Nodes	18,609
Complete Neo4j graph	Directed relationships	41,532
Node label	Factor	9,782
Node label	Paper	7,463
Node label	Gene	1,285
Node label	Level3	45
Node label	Level2	30
Node label	Level1	4
Relationship type	REGULATES	25,202
Relationship type	MENTIONS	12,349
Relationship type	INTERACTS_WITH	2,406
Relationship type	MARKER_OF	1,500
Relationship type	BELONGS_TO	45
Relationship type	PART_OF	30
KGE export	Total triples	19,819
KGE export	Entities	10,660
KGE relation	REGULATES	12,494
KGE relation	MARKER_OF	2,310
KGE relation	INTERACTS_WITH	4,940
KGE relation	BELONGS_TO	45
KGE relation	PART_OF	30

Complete graph statistics were obtained from the Neo4j neuro database. KGE export statistics refer to the deduplicated non-paper training subgraph used for knowledge graph embedding. The difference between complete-graph counts and KGE-export counts reflects relationship merging, removal of Paper nodes and MENTIONS edges, exclusion of non-training evidence attributes and relation-specific preprocessing for marker and STRING-derived interaction triples.

Supplementary Methods 2. Knowledge graph embedding evaluation and microglia prioritization

The KGE input was split using a relation-specific, transductive-safe strategy. BELONGS_TO and PART_OF hierarchy edges were retained in the training set. REGULATES triples were split into training, validation and test sets at an approximate 8:1:1 ratio. MARKER_OF and INTERACTS_WITH triples were split into training and held-out test sets for auxiliary evaluation. Any validation or test triple containing an entity absent from the training set was moved back to the training set. For STRING-derived INTERACTS_WITH edges, unordered gene pairs were split as pairs so that the two directions of the same interaction could not appear in different splits. Final KGE splits contained 17,822 training triples, 383 REGULATES validation triples and 378 REGULATES test triples. The training vocabulary contained 10,660 entities, including 9,782 Factor entities, 799 Gene entities, 45 Level 3 cell entities, 30 Level 2 cell entities and 4 Level 1 cell entities. Models were trained and evaluated across three random seeds. Full-vocabulary filtered evaluation was used for REGULATES link prediction. For cell-type-specific hypothesis generation, a separate type-restricted scoring step ranked Factor entities against a fixed cell-type target. For L3:Microglia, this procedure scored all 9,782 Factor entities. The Microglia prediction results used for Figure 5 were taken from the RotatE seed-42 KGE outputs, included in the public release package under data/kge_results/microglia_regulating_factors.tsv and data/kge_results/microglia_marker_genes.tsv.

Supplementary Table 6. KGE model comparison for REGULATES link prediction

Model	MRR	MRR SD	Hits@1	Hits@1 SD	Hits@3	Hits@3 SD	Hits@10	Hits@10 SD
Model	MRR	MRR SD	Hits@1	Hits@1 SD	Hits@3	Hits@3 SD	Hits@10	Hits@10 SD
ComplEx	0.0218	0.0037	0.0062	0.0016	0.0220	0.0070	0.0454	0.0061
RotatE	0.3750	0.0024	0.3254	0.0029	0.3867	0.0025	0.4705	0.0035
TransE	0.3313	0.0166	0.2769	0.0281	0.3426	0.0078	0.4427	0.0082
TuckER	0.1033	0.0040	0.0335	0.0027	0.0983	0.0114	0.2844	0.0011

Values are mean and standard deviation across three random seeds for full-vocabulary filtered REGULATES evaluation. RotatE was selected for downstream prediction because it achieved the highest overall MRR and Hits@10.

Supplementary Table 7. Direction-specific and auxiliary RotatE evaluation

**Task**	**Evaluation setting**	**MRR**	**Hits@1**	**Hits@3**	**Hits@10**
Task	Evaluation setting	MRR	Hits@1	Hits@3	Hits@10
REGULATES, both directions	Full-vocabulary filtered	0.3750	0.3254	0.3867	0.4705
REGULATES, head prediction	Full-vocabulary filtered	0.3009	0.2848	--	0.3236
REGULATES, tail prediction	Full-vocabulary filtered	0.4490	0.3660	--	0.6173
INTERACTS_WITH, both directions	Gene-pool filtered	0.3089	0.1321	0.4074	0.6521
MARKER_OF retrieval	30-cell macro average	0.2241	--	--	--

REGULATES, INTERACTS_WITH and MARKER_OF values in this table summarize RotatE aggregate performance across three seeds unless otherwise noted. The Microglia prediction panels in Figure 5 use the RotatE seed-42 model selected for representative candidate ranking. MARKER_OF retrieval was evaluated over 30 cells with 320 held-out marker triples and a full gene pool of 799 genes. Macro precision/recall were 0.696/0.370 at top 5, 0.636/0.636 at top 10, 0.442/0.843 at top 20 and 0.310/0.879 at top 30.

Supplementary Table 8. REGULATES test-set entity coverage by hierarchy level

Entity-level coverage of the held-out REGULATES test set (n=378 triples, split seed 42) against the full REGULATES corpus (n=12,494 triples). The test set covered 54 of 66 cell types (81.8%) and 344 distinct regulatory factors. All test entities were present in the training graph, satisfying the transductive constraint.

**Level**	**Entity category**	**In full corpus**	**In test set**	**Coverage (%)**
Level	Entity category	In full corpus	In test set	Coverage (%)
Level 1	Cell types	4	4	100.0
Level 2	Cell types	30	29	96.7
Level 3	Cell types	44	32	72.7
All levels	Cell types	66	54	81.8
---	Regulatory factors	9,782	344	3.5*
L2	Meningeal cell	92	0	---
L3	Central nervous system macrophage	67	0	---
L3	Choroid plexus epithelial cell	53	0	---
L3	Substantia nigra dopaminergic neuron	17	0	---
L3	Hippocampal CA2 pyramidal neuron	14	0	---
L3	arachnoid cell	12	0	---
L3	Dura cell	9	0	---
L3	Chandelier cell	8	0	---
L3	Cortical deep-layer intratelencephalic neuron	8	0	---
L3	Cortical deep-layer corticothalamic neuron	4	0	---
L3	Neuroplacodal cell	4	0	---
L3	Committed oligodendrocyte precursor	1	0	---
L3	Cortical deep-layer near-projecting neuron	1	0	---

*Low factor coverage reflects the long-tailed distribution of regulatory factors (mean 1.28 triples per factor in the full corpus); all 344 test-set factors appear in the training graph. The 13 uncovered cell types are all low-frequency entities (92 triples in the full corpus), indicating that gaps reflect the long-tailed entity distribution rather than a systematic bias of the split.

Supplementary Table 9. REGULATES link-prediction robustness across 5 split seeds

Split-level robustness of RotatE REGULATES link prediction. Five independent 8:1:1 transductive splits were generated with split seeds {1, 2, 3, 4, 5}, while the training seed was fixed at 42 to isolate the effect of data partitioning. The originally reported result (MRR = 0.373, split seed = 42) falls within the observed range (0.327--0.402) and close to the mean. For comparison, varying only the training seed (42/43/44) on the original split yielded MRR = 0.375 0.003 (CV = 0.8%), confirming that training stochasticity is negligible relative to partitioning stochasticity.

**Metric**	**Split 1**	**Split 2**	**Split 3**	**Split 4**	**Split 5**	**Mean SD**	**CV (%)**
Metric	Split 1	Split 2	Split 3	Split 4	Split 5	Mean SD	CV (%)
MRR	0.369	0.402	0.327	0.362	0.386	0.369 0.028	7.6
Hits@1	0.311	0.347	0.273	0.312	0.341	0.317 0.030	9.3
Hits@3	0.376	0.416	0.334	0.368	0.384	0.376 0.030	7.8
Hits@10	0.480	0.507	0.430	0.462	0.478	0.471 0.028	6.0
MRR	0.298	0.326	0.266	0.296	0.318	0.301 0.023	7.8
Hits@10	0.321	0.355	0.290	0.307	0.339	0.322 0.026	8.0
MRR	0.440	0.478	0.388	0.428	0.453	0.437 0.033	7.6
Hits@10	0.639	0.658	0.569	0.617	0.617	0.620 0.033	5.4
Test triples	368	363	383	394	389	---	---

SD = sample standard deviation (ddof = 1); CV = coefficient of variation. All metrics use filtered rank-based evaluation over the full entity vocabulary.

Supplementary Table 10. KGE-prioritized microglia regulator candidates used for in silico knockout

Gene	Novel rank	Global rank	KGE score	Genes tested	Significant DR genes	Top DR gene	Status
Gene	Novel rank	Global rank	KGE score	Genes tested	Significant DR genes	Top DR gene	Status
FMR1	6	763	-5.755	3001	27	MT-RNR1	OK
PTEN	26	787	-6.113	3001	20	AFTPH	OK
FKBP5	38	801	-6.197	3001	18	AFTPH	OK

Candidate regulators were selected from Microglia REGULATES head-prediction rankings after excluding factors with a direct REGULATES edge to Microglia in the training graph. ``Significant DR genes'' indicates genes with significant adjusted P values in the virtual knockout analysis.

Supplementary Methods 3. In silico knockout implementation and released outputs

In silico knockout analysis was performed using the Microglia expression subset from the first-trimester developing human brain single-cell atlas. The expression matrix was downsampled to 500 cells for input loading, and each network construction sampled 300 cells. Genes were filtered from 18,164 detected genes to 3,001 genes during quality control. Wild-type gene regulatory networks were constructed 10 times and combined by tensor decomposition to obtain a consensus network. For each KGE-prioritized candidate gene, virtual knockout was performed by setting the target gene connections to zero, followed by manifold alignment between wild-type and perturbed networks and differential regulation analysis. FMR1, PTEN and FKBP5 were selected because they were high-ranking Microglia regulator candidates without a direct REGULATES edge to Microglia in the KGE training graph. The final analysis identified 27, 20 and 18 significant differentially regulated genes for FMR1, PTEN and FKBP5 knockout, respectively, after multiple-testing adjustment. Full virtual knockout outputs include complete gene-level ranking files, significant differentially regulated gene lists and summary tables. These files are provided as source data with the manuscript release package rather than as local machine paths.

Supplementary Table 11. Top differentially regulated genes from in silico knockout analyses

**Knockout**	**DR gene**	**Distance**	**Z**	**FC**	**P value**	**Adjusted P value**
Knockout	DR gene	Distance	Z	FC	P value	Adjusted P value
FMR1	MT-RNR1	8.647e-07	4.444	108.666	1.920e-25	5.763e-22
FMR1	CYB5R4	7.933e-07	4.303	91.457	1.141e-21	1.712e-18
FMR1	CUL3	7.691e-07	4.253	85.968	1.829e-20	1.830e-17
FMR1	CHASERR	6.700e-07	4.028	65.234	6.650e-16	4.989e-13
FMR1	AFTPH	6.405e-07	3.955	59.613	1.155e-14	6.930e-12
FMR1	B2M	6.362e-07	3.944	58.829	1.720e-14	8.602e-12
FMR1	IER2	6.279e-07	3.923	57.298	3.746e-14	1.606e-11
FMR1	PRKCH	5.884e-07	3.817	50.320	1.306e-12	4.900e-10
FMR1	FMR1	5.340e-07	3.660	41.438	1.217e-10	4.057e-08
FMR1	TET2	5.234e-07	3.628	39.809	2.800e-10	8.404e-08
PTEN	AFTPH	7.625e-07	4.300	90.618	1.743e-21	5.230e-18
PTEN	CYB5R4	5.610e-07	3.831	49.053	2.491e-12	3.738e-09
PTEN	MT-RNR1	5.511e-07	3.804	47.343	5.959e-12	5.961e-09
PTEN	TET2	5.116e-07	3.689	40.801	1.686e-10	1.265e-07
PTEN	ATF6	5.097e-07	3.683	40.499	1.967e-10	1.181e-07
PTEN	PTEN	4.304e-07	3.418	28.873	7.727e-08	3.865e-05
PTEN	CHASERR	3.910e-07	3.266	23.829	1.053e-06	4.513e-04
PTEN	MBD5	3.692e-07	3.175	21.245	4.041e-06	1.516e-03
PTEN	CREB1	3.603e-07	3.136	20.233	6.857e-06	2.286e-03
PTEN	TRIM22	3.529e-07	3.103	19.411	1.054e-05	3.163e-03
FKBP5	AFTPH	9.139e-07	4.736	128.563	8.453e-30	2.537e-26
FKBP5	MT-RNR1	7.536e-07	4.406	87.427	8.747e-21	1.312e-17
FKBP5	MIR99AHG	6.607e-07	4.182	67.199	2.454e-16	2.455e-13
FKBP5	EIF2B3	5.632e-07	3.910	48.829	2.793e-12	2.096e-09
FKBP5	CX3CR1	5.427e-07	3.846	45.338	1.658e-11	9.951e-09
FKBP5	PRKCH	5.125e-07	3.749	40.435	2.033e-10	1.017e-07
FKBP5	ATXN2	5.037e-07	3.720	39.060	4.111e-10	1.762e-07
FKBP5	FKBP5	4.927e-07	3.682	37.371	9.765e-10	3.663e-07
FKBP5	TET2	4.887e-07	3.668	36.758	1.337e-09	4.459e-07
FKBP5	CYB5R4	4.883e-07	3.667	36.709	1.371e-09	4.115e-07

The table lists the top ten significant differentially regulated genes for each KGE-prioritized candidate knockout. Full gene-level ranking files with adjusted P values, significant-gene lists and summaries are provided in the source-data release package.

Supplementary Figure S1. Knowledge graph topological analysis

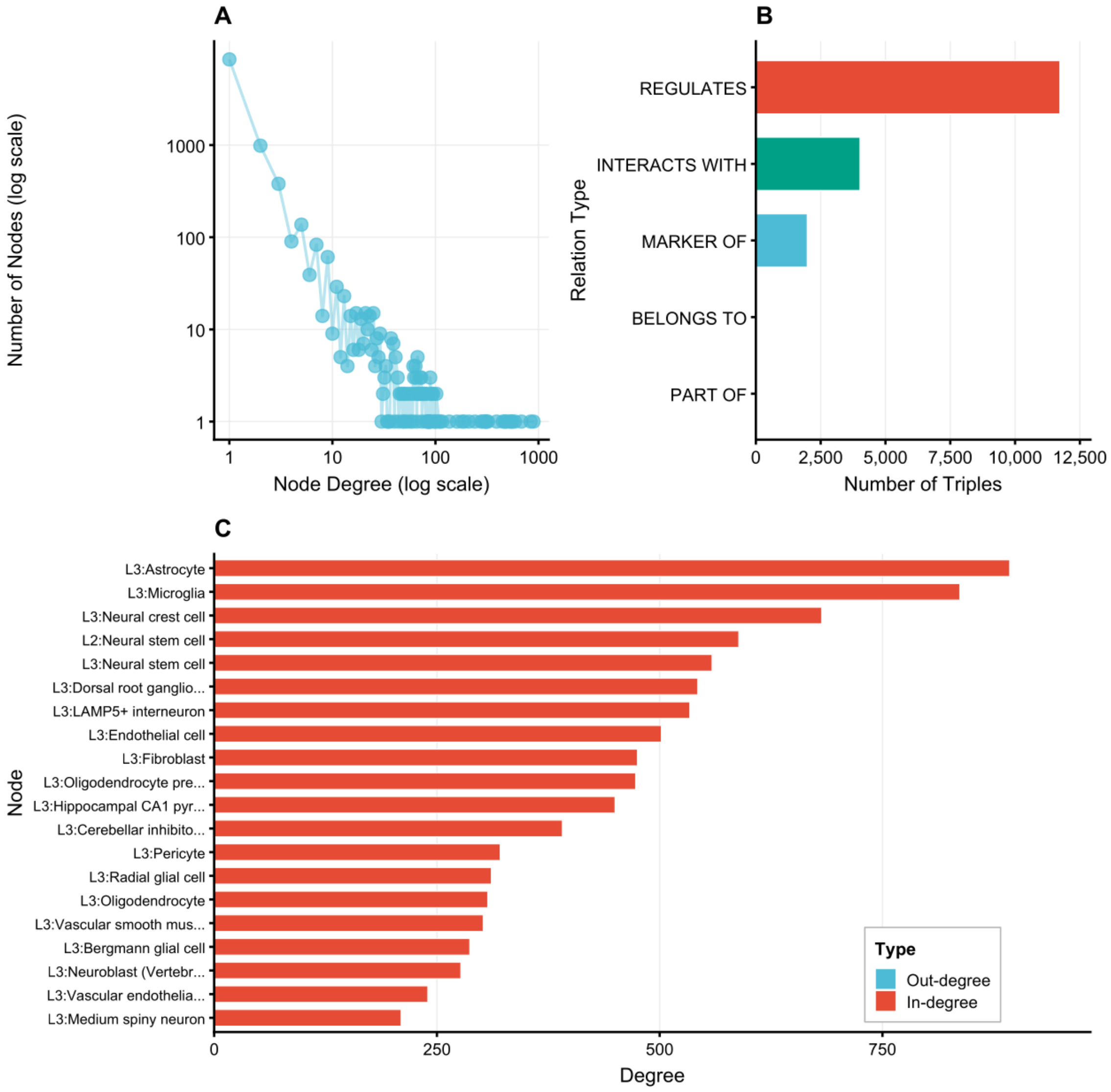

Topological properties of the neural cell knowledge graph. a, Node degree distribution on a log-log scale, showing power-law characteristics typical of biological networks. b, Distribution of relationship types across the knowledge graph. REGULATES relationships dominate, followed by INTERACTS_WITH, MARKER_OF, BELONGS_TO and PART_OF. c, Top 20 hub nodes ranked by total degree. Bars are split into in-degree (coral red) and out-degree (sky blue). High-degree nodes include key regulatory factors and genes central to neural cell regulation. The KGE training subgraph contains 10,660 entities and 17,822 training triples, with an average degree of 3.34.

Supplementary Figure S2. KGE model training and performance comparison

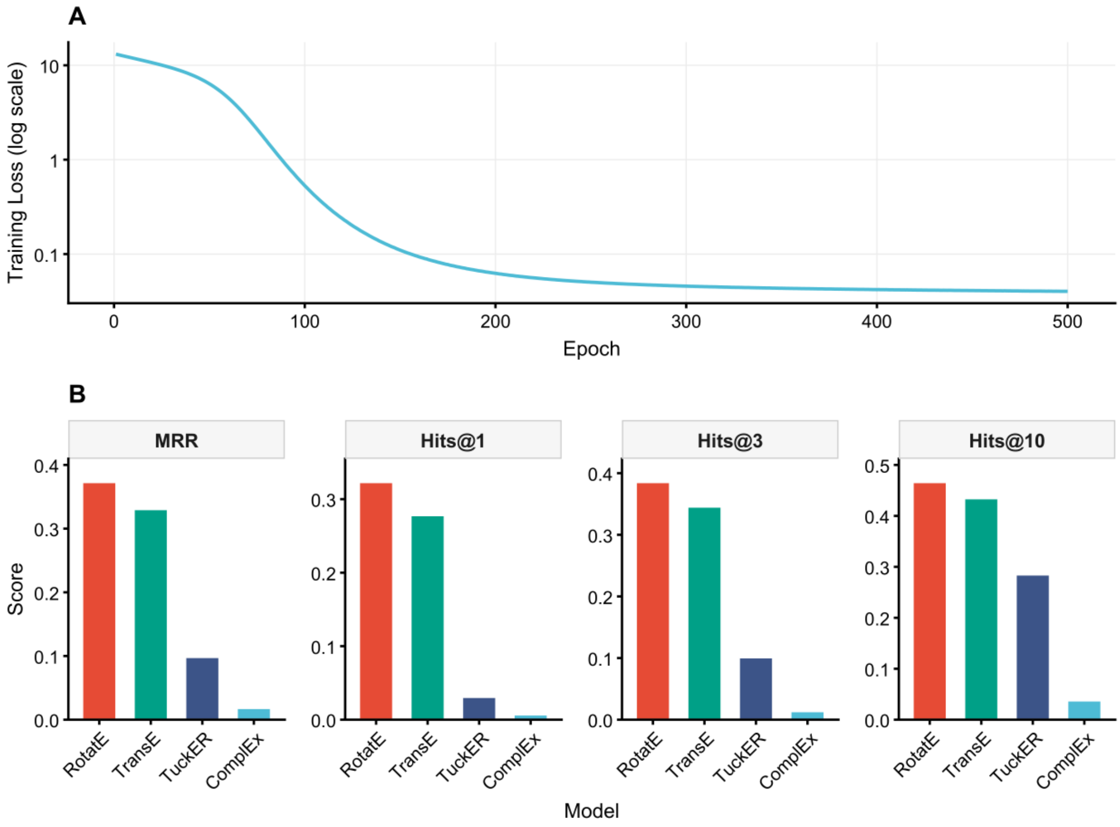

Knowledge graph embedding model training and evaluation. a, Training loss curve for the ComplEx model over 500 epochs (log scale). The loss converges smoothly, indicating stable optimization. b, Performance comparison of four KGE models (RotatE, ComplEx, TransE, TuckER) across four metrics: MRR, Hits@1, Hits@3 and Hits@10. RotatE achieves the best overall performance with MRR = 0.372. All models were trained on 19,819 triples with identical hyperparameters for fair comparison.

Supplementary Figure S3. In silico knockout volcano plots

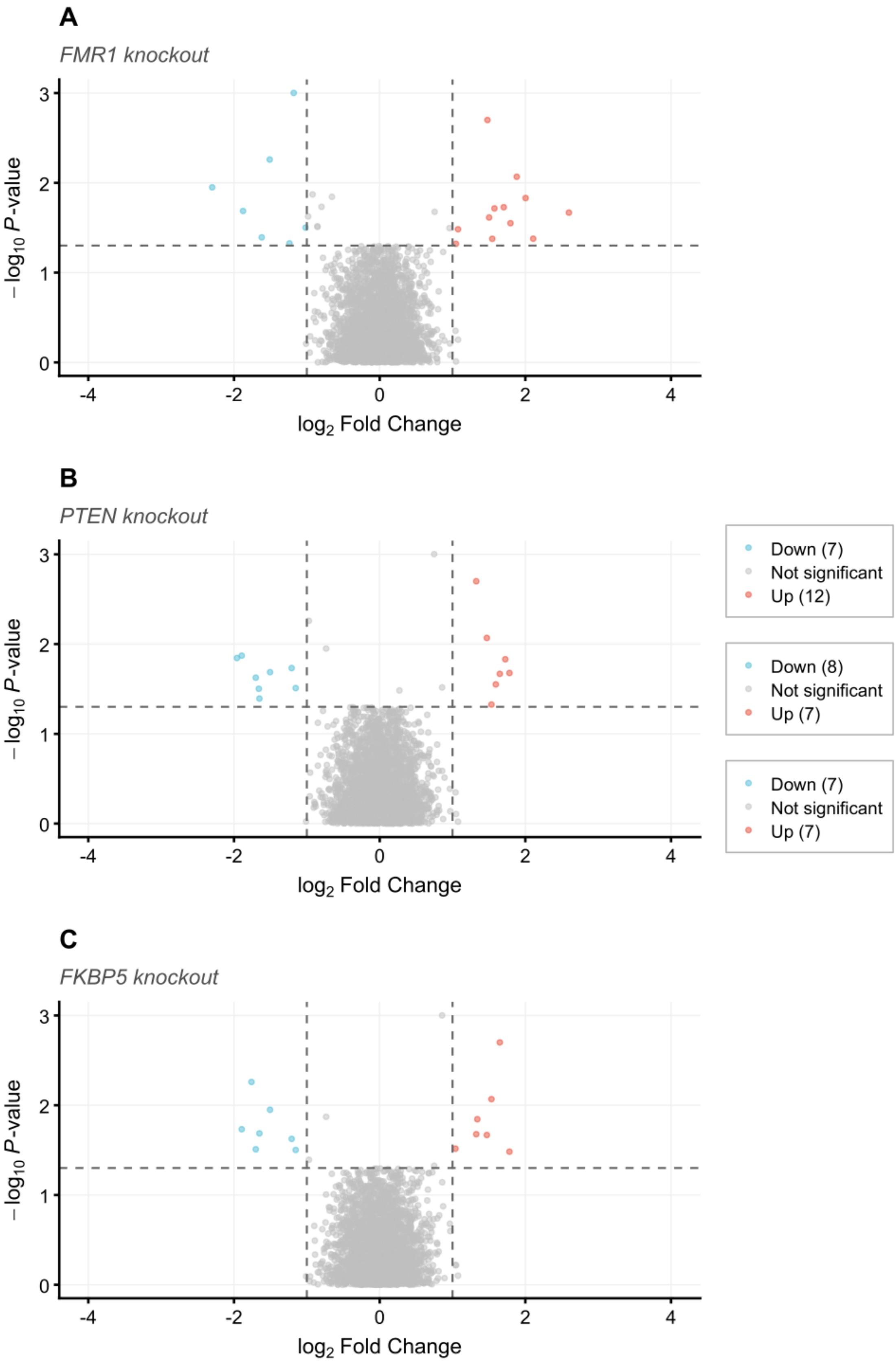

Differential gene expression profiles from in silico knockout analyses. Volcano plots showing differentially expressed genes (DEGs) following virtual knockout of three KGE-prioritized microglia regulators. a, FMR1 knockout induced 27 significant DEGs ($|\log_2 FC| > 1$, $P < 0.05$). b, PTEN knockout induced 20 significant DEGs. c, FKBP5 knockout induced 18 significant DEGs. Upregulated genes are shown in coral red, downregulated genes in sky blue, and non-significant genes in gray. The x-axis represents $\log_2$ fold change and the y-axis represents $-\log_{10}$ adjusted P value. Dashed lines indicate significance thresholds. Recurrent functional themes include mitochondrial metabolism, cytoskeletal remodeling, chromatin regulation and immune signaling. Notably, FKBP5 knockout affected CX3CR1, a canonical microglial marker. Full gene-level rankings and adjusted P values are provided in Supplementary

Supplementary Figure S4. Rule-based inclusion and exclusion workflow for constructing the neural cell hierarchy

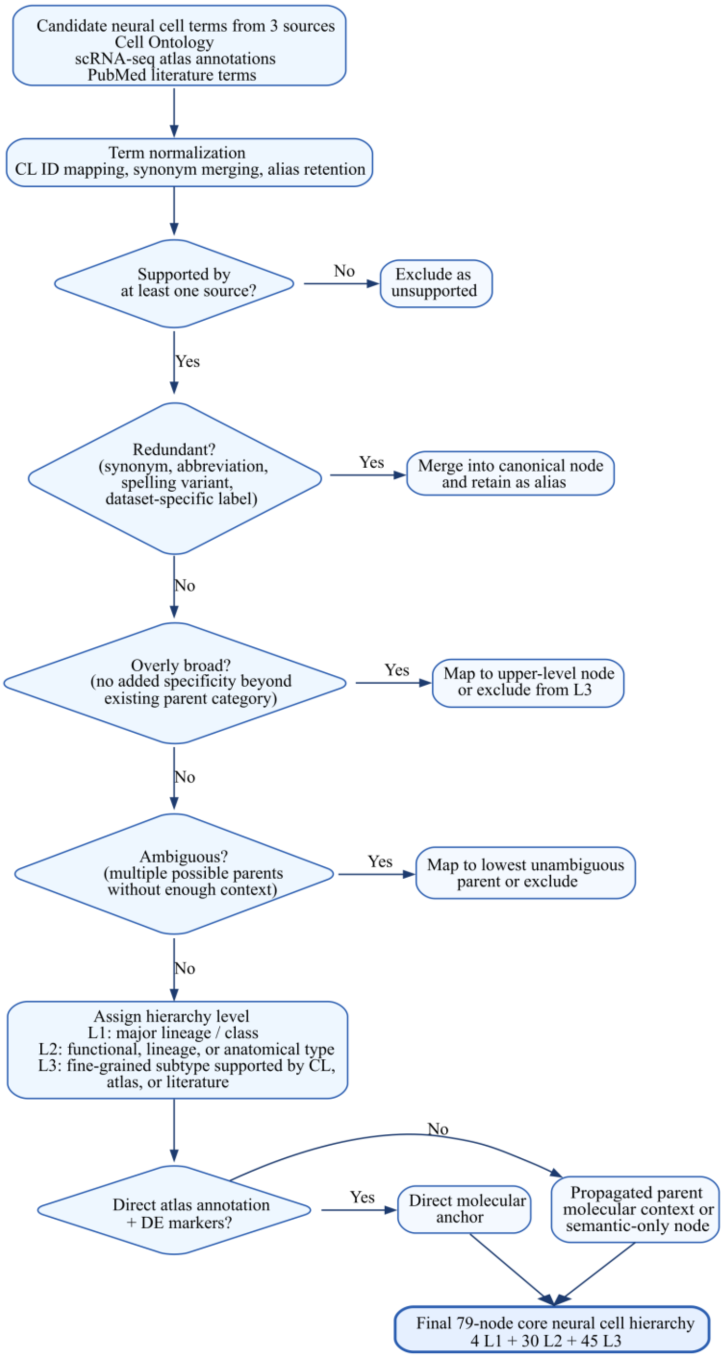

Rule-based inclusion and exclusion workflow for constructing the neural cell hierarchy. Candidate neural cell terms were collected from Cell Ontology, reference single-cell transcriptomic atlases and PubMed-derived literature terms. Terms were normalized by Cell Ontology identifier mapping, synonym merging and alias retention. Candidate terms were then filtered using predefined criteria for redundancy, excessive breadth, ambiguity and evidence support. Included terms were assigned to Level 1, Level 2 or Level 3 according to operational definitions, and each node was annotated with molecular-anchor status. Nodes with direct atlas annotation and differential-expression-supported marker sets were assigned direct molecular anchors, whereas ontology- or literature-supported nodes without direct transcriptomic resolution were retained as semantic anchors with propagated molecular context or no molecular anchor.

Supplementary Data Inventory

The manuscript release package is organized to support reproducibility and reuse. The main data files are:

- data/cell_hierarchy/neural_cells.csv
- data/cell_hierarchy/hierarchy_relations.csv
- data/molecular_fingerprints/marker_genes.tsv
- data/regulatory_triples/regulatory_triples.tsv
- data/regulatory_triples/pubmed_sources.txt
- data/ppi/string_ppi.tsv
- data/kge_splits/train.txt
- data/kge_splits/valid.txt
- data/kge_splits/test.txt
- data/kge_results/model_comparison.csv
- data/kge_results/microglia_regulating_factors.tsv
- data/kge_results/microglia_marker_genes.tsv
- data/virtual_knockout/knockout_summary.csv
- data/virtual_knockout/*_vk_results.csv
- data/virtual_knockout/*_dr_genes.csv
- supplementary/llm_prompts.txt
- supplementary/validation_samples.csv

The package also includes scripts for Neo4j graph construction, KGE splitting, KGE model training, model comparison and task-specific evaluation. The GitHub repository and any permanent archive DOI should be inserted into the Data Availability section after deposition.
